# Inhibiting Skp2 E3 Ligase Suppresses Bleomycin-Induced Pulmonary Fibrosis

**DOI:** 10.3390/ijms19020474

**Published:** 2018-02-06

**Authors:** Masashi Mikamo, Kyoko Kitagawa, Satoshi Sakai, Chiharu Uchida, Tatsuya Ohhata, Koji Nishimoto, Hiroyuki Niida, Sayuri Suzuki, Keiichi I. Nakayama, Naoki Inui, Takafumi Suda, Masatoshi Kitagawa

**Affiliations:** 1 Department of Molecular Biology, Hamamatsu University School of Medicine, 1-20-1 Handayama, Higashi-ku, Hamamatsu, Shizuoka 431-3192, Japan; mas.mik@hama-med.ac.jp (M.M.); kitakyo@hama-med.ac.jp (K.K.); ssakai@hama-med.ac.jp (S.S.); ohhata@hama-med.ac.jp (T.O.); knishi@hama-med.ac.jp (K.N.); niidah@hama-med.ac.jp (H.N.); sayu12160918@hotmail.com (S.S.); 2 Second Division, Department of Internal Medicine, Hamamatsu University School of Medicine, 1-20-1 Handayama, Higashi-ku, Hamamatsu, Shizuoka 431-3192, Japan; inui@hama-med.ac.jp (N.I.); suda@hama-med.ac.jp (T.S.); 3 Advanced Research Facilities & Services, Preeminent Medical Photonics Education & Research Center, Hamamatsu University School of Medicine, 1-20-1 Handayama, Higashi-ku, Hamamatsu, Shizuoka 431-3192, Japan; cuchida@hama-med.ac.jp; 4 Center for Biomedical Research, The Queen’s Medical Center and University of Hawaii, Honolulu, HI 96813, USA; 5 Department of Molecular and Cellular Biology, Medical Institute of Bioregulation, Kyushu University School of Medicine, 3-1-1 Maidashi, Higashi-ku, Fukuoka, Fukuoka 812-8582, Japan; nakayak1@bioreg.kyushu-u.ac.jp; 6 Department of Clinical Pharmacology and Therapeutics, Hamamatsu University School of Medicine, 1-20-1 Handayama, Higashi-ku, Hamamatsu, Shizuoka 431-3192, Japan; 7 Laboratory Animal Facilities & Services, Preeminent Medical Photonics Education & Research Center, Hamamatsu University School of Medicine, 1-20-1 Handayama, Higashi-ku, Hamamatsu, Shizuoka 431-3192, Japan

**Keywords:** Skp2, E3 ligase, p27, bleomycin, pulmonary fibrosis, mouse model, chemical inhibitor, idiopathic pulmonary fibrosis (IPF)

## Abstract

Idiopathic pulmonary fibrosis (IPF) is a progressive disease with poor prognosis and no curative therapies. SCF-Skp2 E3 ligase is a target for cancer therapy, but there have been no reports about Skp2 as a target for IPF. Here we demonstrate that Skp2 is a promising therapeutic target for IPF. We examined whether disrupting *Skp2* suppressed pulmonary fibrosis in a bleomycin (BLM)-induced mouse model and found that pulmonary fibrosis was significantly suppressed in *Skp2*-deficient mice compared with controls. The pulmonary accumulation of fibrotic markers such as collagen type 1 and fibronectin in BLM-infused mice was decreased in *Skp2*-deficient mice. Moreover, the number of bronchoalveolar lavage fluid cells accompanied with pulmonary fibrosis was significantly diminished. Levels of the Skp2 target p27 were significantly decreased by BLM-administration in wild-type mice, but recovered in *Skp2^−/−^* mice. In vimentin-positive mesenchymal fibroblasts, the decrease of p27-positive cells and increase of Ki67-positive cells by BLM-administration was suppressed by *Skp2*-deficency. As these results suggested that inhibiting Skp2 might be effective for BLM-induced pulmonary fibrosis, we next performed a treatment experiment using the Skp2 inhibitor SZL-P1-41. As expected, BLM-induced pulmonary fibrosis was significantly inhibited by SZL-P1-41. Moreover, p27 levels were increased by the SZL-P1-41 treatment, suggesting p27 may be an important Skp2 target for BLM-induced pulmonary fibrosis. Our study suggests that Skp2 is a potential molecular target for human pulmonary fibrosis including IPF.

## 1. Introduction

Idiopathic pulmonary fibrosis (IPF) is a chronic, progressive and deadly diffuse parenchymal lung disorder characterized by destruction of lung architecture with an accumulation of fibroblasts, myofibroblasts, and extracellular matrix (ECM), such as collagen and fibronectin [1]. While the etiology of IPF is not fully understood, it results in impaired pulmonary function ultimately leading to respiratory failure. IPF patients have a poor prognosis, with a median survival of 2–4 years [2].

Until recently, there was no effective pharmacological IPF treatment, and lung transplantation was the only treatment that had been shown to improve prognosis [3]. Thus, there is urgent need to develop more effective agents.

It is currently believed that IPF results from an interaction between exogenous and endogenous factors. Environmental exposure, including cigarette smoking, metal and wood dust, farming, viruses and stone debris, have been observed to increase the risk of IPF [4,5]. Several candidate genes involved in the onset of IPF have also been reported, but molecularly, little has been elucidated.

The bleomycin (BLM)-induced mouse pulmonary fibrosis model is the most popular model used for evaluating the potential effects of therapeutics and investigating mechanisms of IPF development [6]. BLM is an antitumor agent that induces double strand DNA breaks to tumor cells, leading to apoptotic cell death. BLM also severely induces lung injury and fibrosis. In early stages, BLM-administration induces pulmonary epithelial cell death, and in middle stages, induces the infiltration of neutrophils, macrophages and lymphocytes. Then, activated fibroblasts produce ECM proteins such as collagens and fibronectin and develop typical fibrosis about two weeks after BLM-administration [6].

We have previously reported that unilateral ureteral obstruction-induced chronic renal fibrosis in mice is suppressed by S-phase kinase-associated protein 2 (*Skp2*)-deficiency [7]. Skp2 is an F-box protein that plays critical roles in the Skp1/Cullin 1/F-box (SCF)-Skp2 ubiquitin ligase complex [8,9]. SCF-Skp2 targets and ubiquitylates several growth inhibitory proteins such as cyclin-dependent kinase (CDK) inhibitors (p27, p21, p57) and tumor suppressor proteins (p130 and Tob1) for proteasome-mediated degradation [8,10,11]. In the chronic renal fibrosis model, our data suggested that Skp2 promotes the tubular epithelial cell proliferation required for tubule dilatation that is accompanied by renal injury progression via p27-degradation [7,12]. Based on these findings, we speculated that Skp2 might also contribute to pulmonary fibrosis and be a potential molecular target for IPF therapy. To test this hypothesis, we investigated whether *Skp2*-deficiency affects fibrotic progression in the BLM-induced mouse pulmonary fibrosis model. Moreover, we evaluated whether a small molecule Skp2 inhibitor could be a potential therapy for IPF.

## 2. Results

### 2.1. BLM-Induced Lung Fibrosis Was Suppressed in Skp2-Deficient Mice

We evaluated whether Skp2 is a potential molecular target for IPF using the BLM-induced pulmonary fibrosis mouse model. As shown in Supplementary Figure S1, pulmonary fibrosis began one week after BLM-injection and reached a maximum level after 3–4 weeks, as evaluated by Masson trichrome (MT) staining and type 1 collagen 1 (COL1A1) immunostaining. Because fibrotic changes began one week after BLM-injection and were more evident and extensive after two weeks, we evaluated the effects of *Skp2*-deficiency at a relatively early stage of pulmonary fibrosis progression using *Skp2*-deficient mice two weeks after BLM-injection.

**Figure 1:**
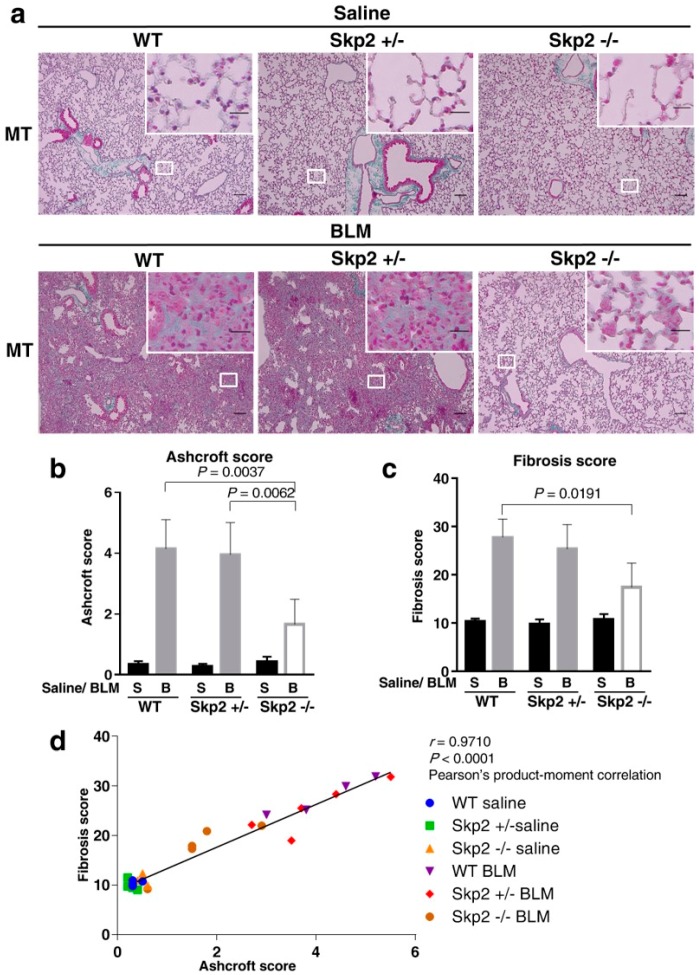
Bleomycin-induced pulmonary fibrosis was suppressed in *Skp2*-deficient mice: *Skp2*^+/+^ (WT), *Skp2*^+/−^ and *Skp2*^−/−^ mice were intratracheally infused 2 mg/kg bleomycin (BLM) (*n* = 4:5:5, respectively) or saline (*n* = 4:5:4, respectively). Two weeks after BLM administration, bronchoalveolar lavage fluids were obtained. Then the mice were sacrificed and lung tissues were harvested. The paraffin-embedded lung tissues were subjected to hematoxylin-eosin (HE) staining (Supplementary Figure S2a) and Masson’s trichrome (MT) staining. (**a**) Representative lung images of MT staining in WT, *Skp2*^+/−^ and *Skp2*^−/−^ mice infused with saline (upper panels) or BLM (lower panels). The scale bar indicates 100 μm in the low magnification images and 20 μm in the high magnification images. (**b**,**c**) BLM-induced pulmonary fibrosis was suppressed in *Skp2*^−/−^ mice. Degrees of pulmonary fibrosis were graded and evaluated by the Ashcroft method (**b**) as described in Materials and Methods. Fibrotic regions stained green in MT staining were measured and calculated as the fibrosis score (**c**) as described in Materials and Methods. (**d**) Pearson’s product–moment correlation coefficient showed a significant correlation between the scores from the two evaluation methods.

As shown in Supplementary Figure S2a, hematoxylin and eosin (HE) staining of lung sections indicated that saline-infused mice showed approximately the same normal morphology of lung tissues among wild-type (WT), *Skp2^+/−^* and *Skp2^−/−^* mice. There were apparent lung injury features such as thickening of the alveolar walls, inflammatory cell infiltration, focal regions of damaged alveolar structure, and distorted pulmonary architecture in the lung interstitium in BLM-infused mice lungs indicated by HE staining and ECM accumulation indicated by the green area in MT staining in both WT and *Skp2^+/−^* mice, whereas these fibrotic features were clearly reduced in *Skp2^−/−^* mice (Figure 1a and Supplementary Figure S2a).

To compare the degree of pulmonary fibrosis, we evaluated two different scores: the Ashcroft score and the fibrosis score. As described in Materials and Methods, microscopic HE-stained tissue images were graded according to the Ashcroft method (Figure 1b) [13]. Moreover, green fibrotic areas versus red non-fibrotic areas after MT staining were quantified using ImageJ software, and then their ratios were calculated as the fibrosis score (Figure 1c). As shown in Figure 1b, BLM-infused *Skp2^−/−^* mice lungs showed significantly lower Ashcroft scores than BLM-infused WT and *Skp2^+/−^* mice lungs (WT vs. *Skp2^−/−^*: *p* = 0.0037, *Skp2^+/−^* vs. *Skp2^−/−^*: *p* = 0.0062). Moreover, as shown in Figure 1c, BLM-infused *Skp2^−/−^* mice lungs showed significantly lower fibrosis scores compared with BLM-infused WT mice lungs (WT vs. *Skp2^−/−^*: *p* = 0.0191). To evaluate whether the Ashcroft and fibrosis scores were correlated, the correlation between these two scores for each sample were compared. As shown in Figure 1d, the Ashcroft and fibrosis scores were significantly correlated using Pearson’s product–moment correlation (*r* = 0.9710, *p* < 0.0001). This result supports the accuracy of these evaluation methods, and therefore, strongly suggested that BLM-induced pulmonary fibrosis was suppressed in *Skp2*-deficient mice.

### 2.2. Skp2-Deficiency Suppressed the Accumulation of Fibrosis Markers in the BLM Model

**Figure 2:**
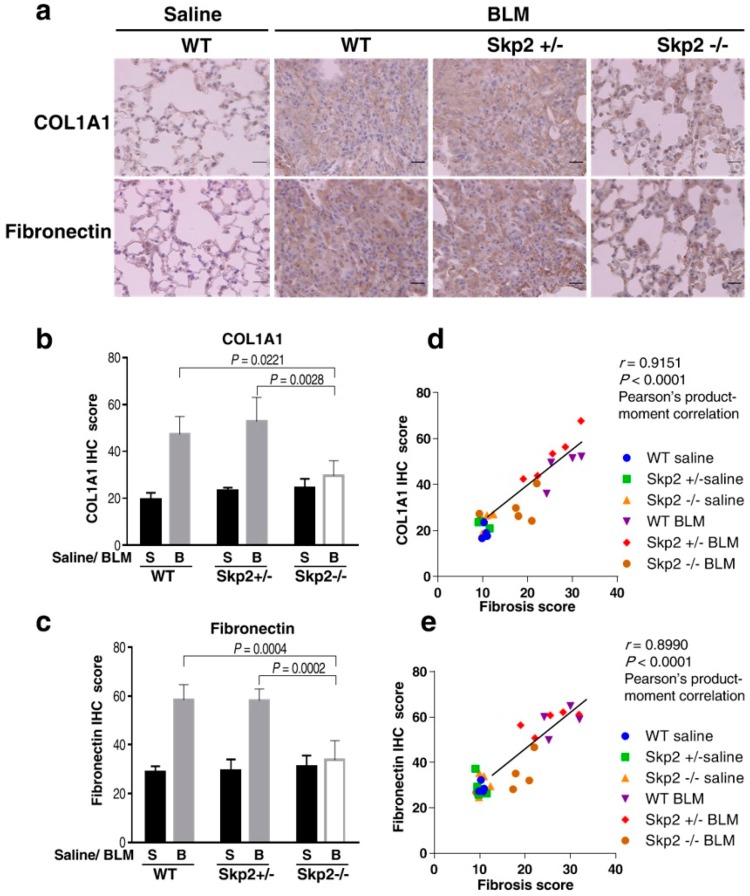
*Skp2*-deficiency suppressed the accumulation of fibrosis markers in BLM model mice: (**a**) Representative 3,3′-diaminobenzidine (DAB)-colored images of type 1 collagen 1 (COL1A1) and fibronectin immunostaining in BLM-infused mice lung. Paraffin-embedded lung tissues from BLM-infused *Skp2^+/+^* (WT), *Skp2^+/−^* and *Skp2^−/−^* mice were subjected to immunostaining with anti-COL1A1 antibody (upper panel) or anti-fibronectin antibody (lower panel). The scale bar indicates 20 μm. (**b**,**c**) The elevated COL1A1 expression (**b**) and fibronectin expression (**c**) seen in the BLM model was decreased in *Skp2^−/−^* mice. Intensities of the DAB-immunostaining with anti-COL1A1 (**b**) or anti-fibronectin (**c**) antibody were scored and evaluated by Tukey’s test as described in Materials and Methods. (**d**,**e**) Pearson’s product–moment correlation coefficient showed a significant correlation between fibrosis scores and COL1A1 expression (**d**) or fibronectin expression (**e**).

We further investigated the effects of *Skp2*-deficiency on the expression of fibrosis markers in BLM-induced fibrosis by performing immunohistochemistry on the lung tissues used in Figure 1 using antibodies against COL1A1 and fibronectin. BLM-infused WT mice showed accumulations of both COL1A1 and fibronectin, while BLM-infused *Skp2^−/−^* mice lungs showed weak COL1A1 and fibronectin staining compared with BLM-infused WT and *Skp2^+/−^* lungs (Figure 2a). Moreover, we measured staining intensities using Image J and statistically evaluated these results as described in Materials and Methods. These results showed that BLM-infused *Skp2^−/−^* mice lungs showed significantly lower COL1A1 staining than BLM-infused WT and *Skp2^+/−^* mice lungs (WT vs. *Skp2^−/−^*: *p* = 0.0221, *Skp2^+/−^* vs. *Skp2^−/−^*: *p* = 0.0028) (Figure 2b). Moreover, BLM-infused *Skp2^−/−^* mice lungs also showed significantly reduced fibronectin staining compared with BLM-infused WT and *Skp2^+/−^* mice lungs (WT vs. *Skp2^−/−^*: *p* = 0.0004, *Skp2^+/−^* vs. *Skp2^−/−^*: *p* = 0.0002) (Figure 2c).

We then evaluated whether COL1A1 and fibronectin accumulations correlated with the fibrosis scores in Figure 2d,e, respectively. Both the COL1A1 and fibronectin staining intensities were significantly correlated with the fibrosis scores in Pearson’s product–moment correlation (COL1A1 vs. fibrosis score: *r* = 0.9151, *p* < 0.0001; fibronectin vs. fibrosis score: *r* = 0.8990, *p* < 0.0001). Therefore, the accumulation of fibrotic markers was significantly correlated with fibrosis scores. These results indicated that *Skp2*-deficiency suppressed the accumulation of fibrosis markers in the BLM model.

Additionally, as shown in Supplementary Figure S3, genotypes of the BLM model correlated with Skp2 mRNA expression but not p27 mRNA expression in lungs. This result is reasonable, as Skp2 carries out the degradation of p27 protein as a protein ubiquitin ligase irrespective of the expression of p27 mRNA. mRNA expressions of COL1A1 and fibronectin had a tendency to be inhibited in *Skp2^−/−^* mice and these results are consistent with the immunohistochemistry result in Figure 2.

### 2.3. Effects of Skp2-Deficiency on Bronchoalveolar Lavage Fluid Cells in the BLM Mouse Model

**Figure 3:**
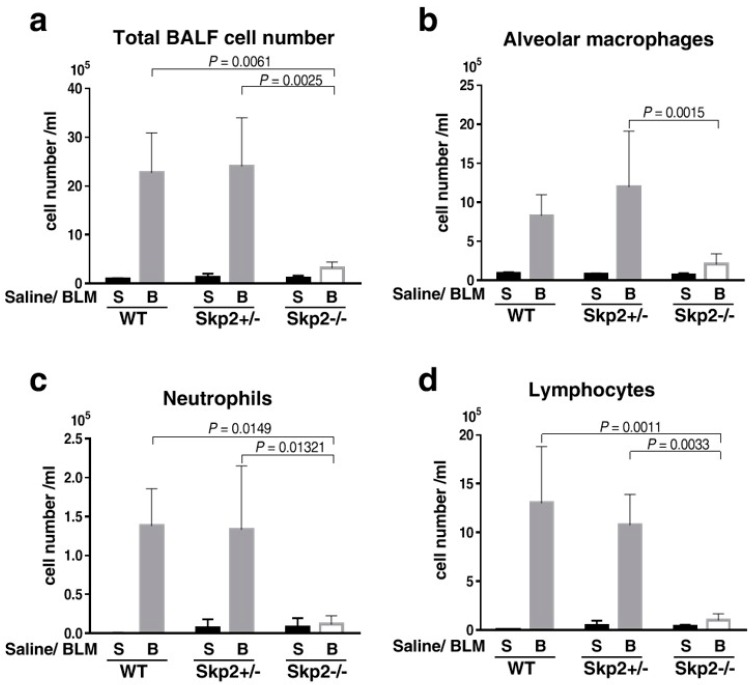
Effects of *Skp2*-deficiency on bronchoalveolar lavage fluid cells in BLM model mice: Bronchoalveolar lavage fluids (BALF) were obtained from BLM- or saline-infused *Skp2^+/+^* (WT), *Skp2^+/−^* and *Skp2^−/−^* mice. (**a**) The total number of BALF cells were counted and evaluated by Tukey’s test. (**b**–**d**) The number of pulmonary alveolar macrophages (**b**), neutrophils (**c**) and lymphocytes (**d**) in BLM model mice were counted and evaluated by Tukey’s test. The number of these cells in BLM-infused mice were significantly suppressed by *Skp2*-deficiency.

BLM-induced lung fibrosis is accompanied by an accumulation of bronchoalveolar lavage fluid (BALF) cells. Therefore, we evaluated the accumulation of BALF cells in the BLM model. As shown in Figure 3a, the total number of BALF cells was increased after BLM-administration; however, the total number of BALF cells from BLM-infused *Skp2^−/−^* mice was significantly decreased compared with BLM-infused WT and *Skp2^+/−^* mice (WT vs. *Skp2^−/−^*: *p* = 0.0061, *Skp2^+/−^* vs. *Skp2^−/−^*: *p* = 0.0025). Moreover, the numbers of alveolar macrophages, neutrophils and lymphocytes in BALF were reduced in BLM-infused *Skp2^−/−^* mice compared with BLM-infused WT and *Skp2^+/−^* mice (Figure 3b–d). Figure 1, Figure 2 and Figure 3 strongly suggest that BLM-induced pulmonary fibrosis was suppressed in *Skp2*-deficient mice.

### 2.4. The Effects of Skp2-Deficiency on p27 Levels in BLM-Induced Lung Fibrosis

Many studies indicated that the CDK inhibitor p27*^Kip1^* (p27) is a main target for SCF-Skp2 E3 ligase. Therefore, we performed p27 immunostaining in the murine lung sections to determine the involvement of p27 as a Skp2 target during the progression of BLM-induced lung fibrosis. As shown in Supplementary Figure S4, the 3,3’-diaminobenzidine (DAB) intensities of p27-staining in saline-infused WT mice were significantly decreased in BLM-infused WT mice (*p* = 0.0362). In contrast, p27 staining intensities in *Skp2^−/−^* mice did not decrease following BLM infusion. This result suggested that the decreased p27 expression in BLM-induced pulmonary fibrosis was recovered in *Skp2*-deficient mice.

### 2.5. Skp2-Deficiency May Suppress the BLM-Induced Increase in Mesenchymal Fibroblasts

**Figure 4:**
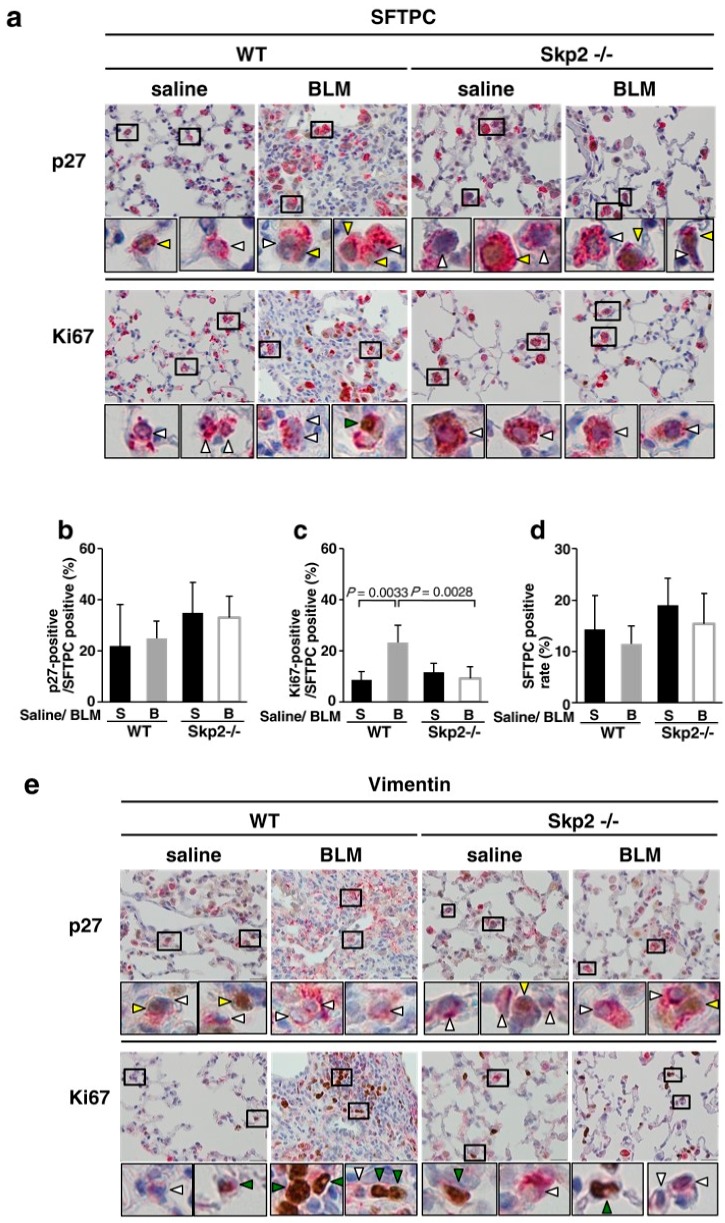

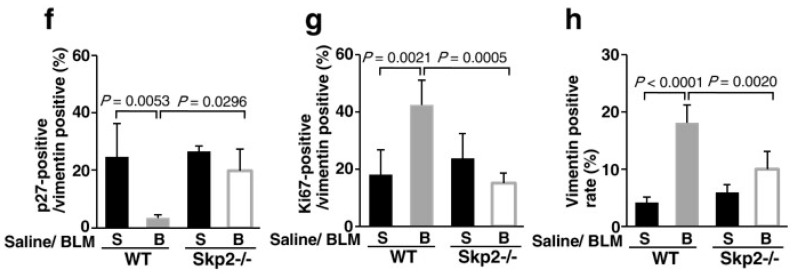
Effects of *Skp2*-deficiency on p27-expression and proliferation of type II alveolar epithelial cells and mesenchymal fibroblasts in BLM-induced pulmonary fibrosis: Paraffin-embedded lung tissues from *Skp2^+/+^* (WT) and *Skp2^−/−^* mice infused with saline or BLM as indicated in Figure 1 were subjected to immunostaining with anti-p27 antibody or anti-Ki67 antibody using DAB and with anti-surfactant protein C (SFTPC) antibody for type II alveolar epithelial cells (AECII) (**a**–**d**) or anti-vimentin antibody for mesenchymal fibroblasts (**e**–**h**) using alkaline phosphatase-mediated staining. (**a**) Representative double immunostaining images with anti-SFTPC antibody and anti-p27 antibody or anti-Ki67 antibody in saline-infused and BLM-infused mice lung. In the high magnification images, yellow and white arrowheads indicate p27-positive and -negative AECII cells, respectively. Green and white arrowheads indicate Ki67-positive and -negative AECII cells, respectively. Additional representative images are indicated in Supplementary Figure S5. The scale bar indicates 20 μm. (**b**) Ratios of p27-positive cells in SFTPC-positive cells. (**c**) Ratios of Ki67-positive cells in SFTPC-positive cells. (**d**) The abundance of SFTPC-positive AECII cells. (**e**) Representative double immunostaining images with anti-vimentin antibody and anti-p27 antibody or anti-Ki67 antibody in saline-infused and BLM-infused mice lung. In the high magnification images, yellow and white arrowheads indicate p27-positive and -negative mesenchymal fibroblasts, respectively. Green and white arrowheads indicate Ki67-positive and -negative mesenchymal fibroblasts, respectively. Additional representative images are indicated in Supplementary Figure S5. The scale bar indicates 20 μm. (**f**) Ratios of p27-positive cells in vimentin-positive cells. (**g**) Ratios of Ki67-positive cells in vimentin-positive cells. (**h**) The abundance of vimentin-positive mesenchymal fibroblasts.

Double immunostaining experiments to identify p27-positive cells were performed using the paraffin-embedded samples from Figure 1. To identify type II alveolar epithelial cells (AECII) and mesenchymal fibroblasts, antibodies against surfactant protein C (SFTPC) and vimentin were used, respectively. Figure 4a–d indicated that Skp2 may partially participate in the cell proliferation of AECII, but this might not be sufficient to affect the abundance of AECII cells. In vimentin-positive mesenchymal fibroblasts, the decrease of p27-positive cells and increase of Ki67-positive cells by BLM-administration was suppressed by *Skp2*-deficency (Figure 4e–h). Moreover, the increase of mesenchymal fibroblasts by BLM-administration was suppressed by *Skp2*-deficency (Figure 4e,g). These results suggest that Skp2 may participate in the BLM-induced increase of mesenchymal fibroblasts via the Skp2/p27 axis.

### 2.6. The Skp2 Inhibitor SZL-P1-41 Suppressed BLM-Induced Lung Fibrosis

The results in Figure 1, Figure 2 and Figure 3 strongly suggested that Skp2 is required for the progression of BLM-induced pulmonary fibrosis. Therefore, we speculated that inhibiting the SCF-Skp2 E3 ligase activity through a small molecule compound might suppress pulmonary fibrosis. Several types of chemical Skp2 inhibitors were explored as therapeutic drugs for malignant cancers, whereas there are no reports about the application of Skp2 inhibitors against pulmonary fibrosis. Chan et al. reported that SZL-P1-41 inhibits the interaction between Skp2 and Skp1 and shows inhibitory activity against SCF-Skp2 but not against either SCF-Fbw7 or SCF-β-TrCP [14]. SZL-P1-41 also inhibits Skp2-dependent cell growth in cancer cell lines in vitro. Moreover, SZL-P1-41 (80 mg/kg, daily injection) shows anti-tumor activity in mouse xenograft models [14].

**Figure 5:**
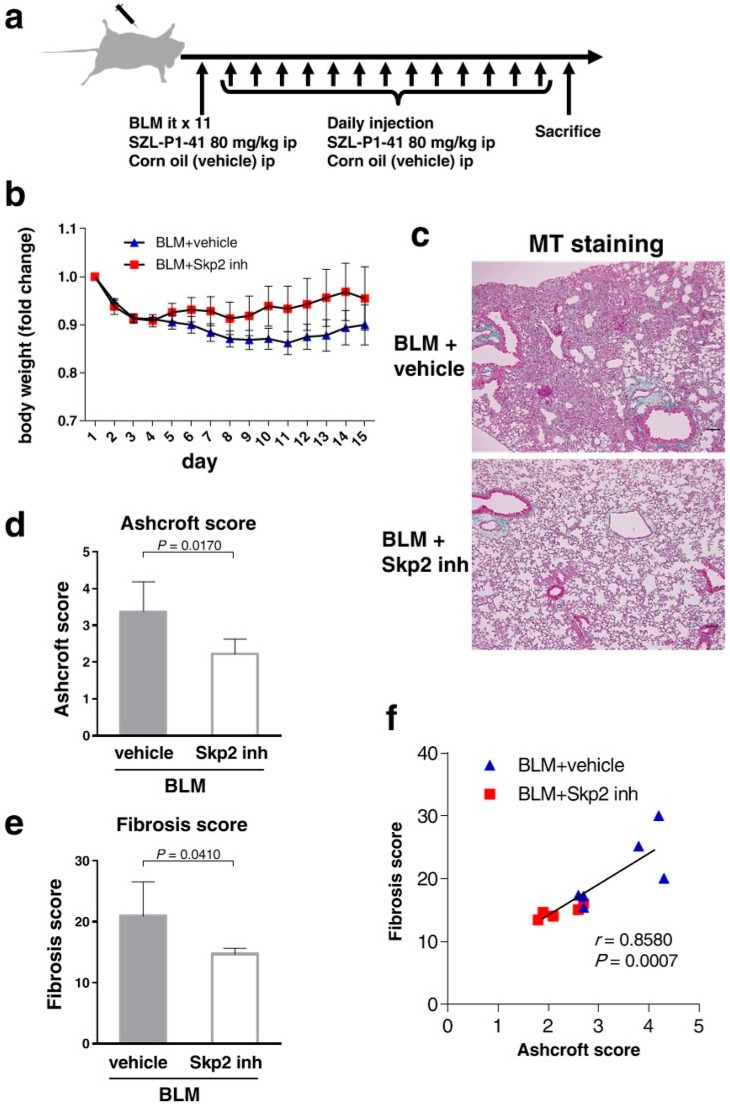
SZL-P1-41 inhibited pulmonary fibrosis in BLM model mice: (**a**) Wild type mice were intratracheally infused BLM (2 mg/kg). The Skp2 inhibitor SZL-P1-41 (80 mg/kg) (*n* = 5) or corn oil (vehicle) (*n* = 6) was injected intraperitoneally daily from day 1 to day 14. (**b**) Body weights of mice in the different treatment groups; SZL-P1-41 (red) and vehicle (blue). (**c**) Two weeks after BLM treatment, BALF (Supplementary Figure S6) and lung tissues were obtained from the mice. The paraffin-embedded lung tissues were analyzed by HE (Supplementary Figure S2b) and MT (**c**) staining. The scale bar indicates 100 μm. (**d**–**f**) BLM-induced pulmonary fibrosis was suppressed in SZL-P1-41-treated mice. Degrees of pulmonary fibrosis were scored and evaluated by Ashcroft (**d**) and fibrosis (**e**) scores. (**f**) Pearson’s product–moment correlation coefficient showed a significant correlation between the scores from the two evaluation methods.

Based on these reports, we investigated whether BLM-induced lung fibrosis was suppressed by SZL-P1-41 treatment. Wild type mice were intratracheally infused BLM (2 mg/kg). The Skp2 inhibitor SZL-P1-41 (80 mg/kg) (*n* = 5) or corn oil (vehicle) (*n* = 6) was injected intraperitoneally daily from day one to day 14 (Figure 5a). Because of the lung injury by BLM, body weights gradually decreased in vehicle-injected mice (Figure 5b). Interestingly, the decreased body weight in BLM-infused mice was recovered by SZL-P1-41 treatment, supporting the conclusion that Skp2 inhibition attenuates the progression of pulmonary fibrosis.

As shown in Figure 5c, vehicle-injected BLM mice showed apparent lung injury features such as thickening of the alveolar walls, focal regions of damaged alveolar structure, and distorted pulmonary architecture in the lung interstitium by HE staining and ECM accumulation indicated by green in MT staining.

These fibrotic features in the lungs were suppressed by SZL-P1-41 treatment. Both the Ashcroft score (Figure 5d) and fibrosis score (Figure 5e) were significantly lower in SZL-P1-41-treated mice than in vehicle-treated mice (*p* = 0.0170 and *p* = 0.0410, respectively). Moreover, the Ashcroft and fibrosis scores were significantly correlated in the Pearson’s product–moment correlation (*r* = 0.8580, *p* = 0.0007) (Figure 5f), highlighting the efficacy of these evaluations. These results suggested that BLM-induced pulmonary fibrosis was suppressed by the Skp2 inhibitor.

**Figure 6:**
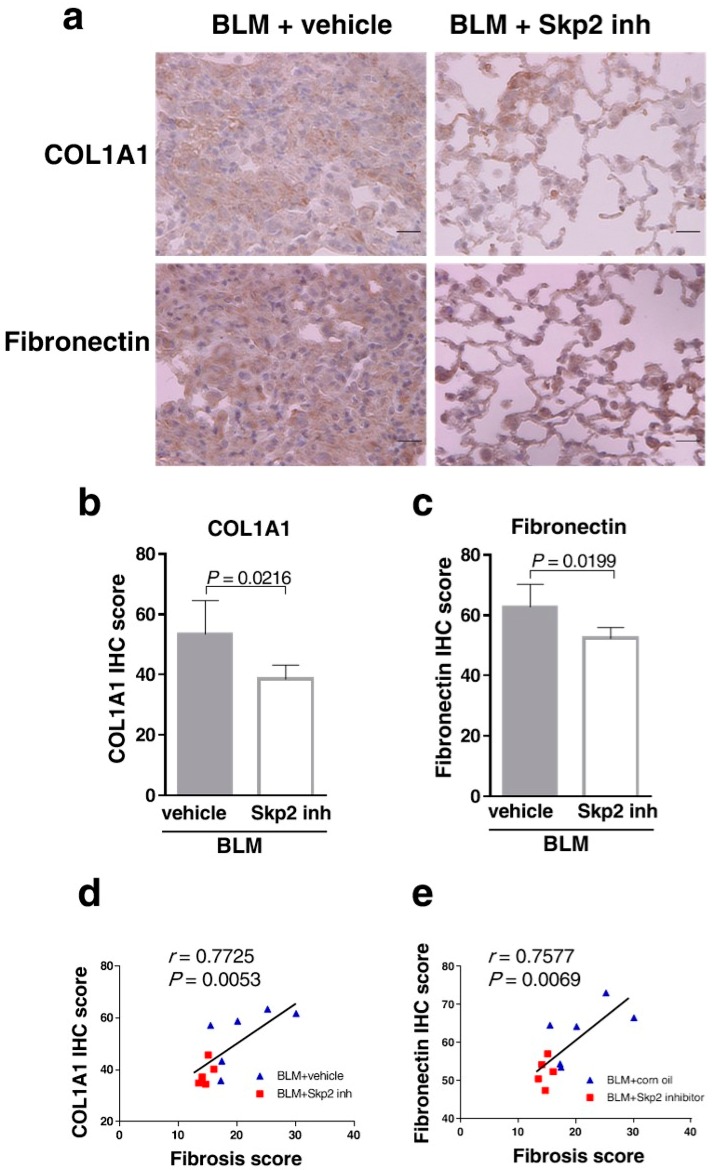
SZL-P1-41 suppressed the expression of fibrosis markers in BLM model mice: (**a**) Representative DAB-colored images of COL1A1 and fibronectin immunostaining in SZL-P1-41-treated BLM model mice. The scale bar indicates 20 μm. (**b**,**c**) The BLM-induced COL1A1 expression (**b**) and fibronectin expression (**c**) were suppressed by SZL-P1-41 treatment. Intensities of anti-COL1A1 (**b**) or anti-fibronectin (**c**) immunostaining were scored and evaluated by Student’s *t*-test as described in Materials and Methods. (**d**,**e**) Pearson’s product–moment correlation coefficient showed a significant correlation between fibrosis scores and COL1A1 expression (**d**) or fibronectin expression (**e**).

Moreover, we confirmed that the Skp2 inhibitor suppressed pulmonary fibrosis by evaluating the expression of fibrosis markers. SZL-P1-41-treated mice lungs showed significantly reduced COL1A1 and fibronectin staining compared with vehicle-treated mice lungs (*p* = 0.0216 and *p* = 0.0199, respectively) (Figure 6a–c).

The staining intensities of both COL1A1 and fibronectin were significantly correlated with fibrosis scores in the Pearson’s product–moment correlation (COL1A1 vs. fibrosis score: *r* = 0.7725, *p* = 0.0053; fibronectin vs. fibrosis score: *r* = 0.7577, *p* = 0.0069) (Figure 6d and e, respectively). Therefore, accumulations of fibrosis markers were significantly correlated with fibrosis scores. These results indicate that SZL-P1-41 suppressed BLM-induced fibrosis accompanied by the accumulation of fibrosis markers.

Next, we found the total number of cells in BALF decreased in SZL-P1-41-treated mice compared with vehicle-treated controls (Supplementary Figure S6a). The numbers of lymphocytes in BALF were significantly reduced in SZL-P1-41-treated mice, whereas those of both alveolar macrophages and neutrophils did not change (Supplementary Figure S6b–d).

The paraffin-embedded samples of Figure 5 were subjected to double immunostaining as described in Figure 4 (Supplementary Figure S7a). In SFPTC cells, p27-positive cells were increased and Ki67-positive cells were decreased, by SZL-P1-41 treatment; however, this decreased cell proliferation did not correlate with the number of AECII cells (Supplementary Figure S7b–d). In vimentin-positive mesenchymal fibroblasts, p27-positive cells showed a tendency to be increased by SZL-P1-41 treatment and Ki67-positive cells showed a tendency to be decreased by the treatment (Supplementary Figure S7e,f). Moreover, the abundance of mesenchymal fibroblasts was suppressed by SZL-P1-41 treatment (Supplementary Figure S7g). This result is consistent with Figure 4. The decrease of mesenchymal fibroblast may be caused by the suppression of cell proliferation by Skp2-inhibition.

**Figure 7:**
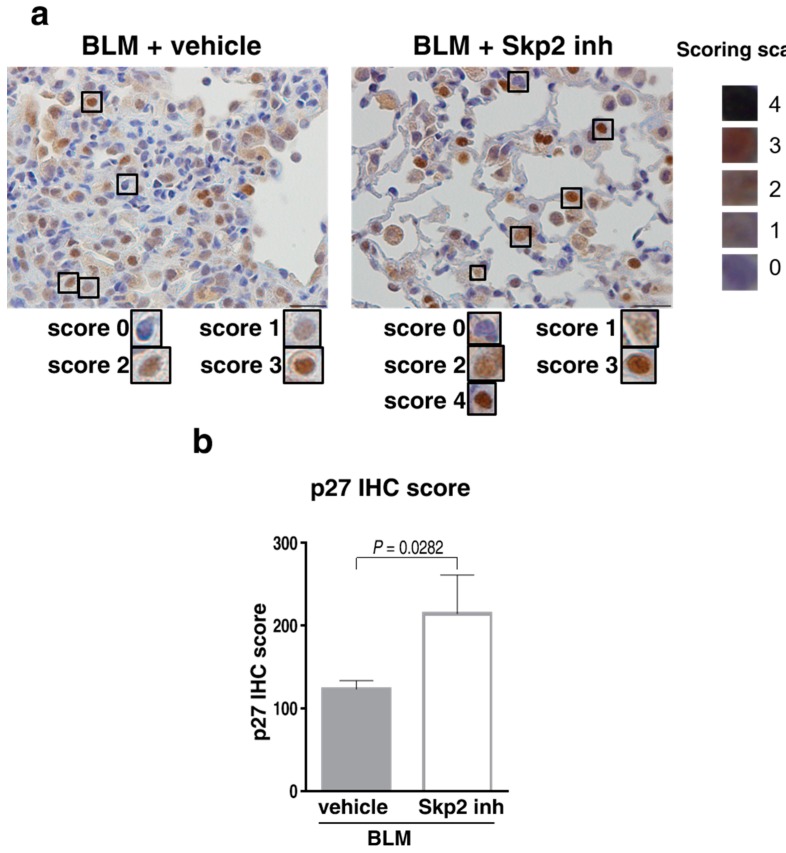
Effects of SZL-P1-41 treatment on p27 levels in BLM-induced lung fibrosis: (**a**) Representative DAB-colored images of anti-p27 immunostaining. Paraffin-embedded lung tissues from BLM-infused WT mice treated with vehicle (left panel) or SZL-P1-41 (right panel) as indicated in Figure 5 were subjected to immunostaining with anti-p27 antibody. The scale bar indicates 20 μm. (**b**) p27 levels were significantly increased in SZL-P1-41-treated BLM model mice. DAB intensities of anti-p27 immunostaining were scored and evaluated by Student’s t-test as described in Materials and Methods.

Moreover, as shown in Figure 7, p27 staining intensities were significantly increased in SZL-P1-41-treated, BLM-infused mice lungs compared with vehicle-treated mice lungs (*p* = 0.0282). The result suggested that SZL-P1-41 inhibited p27 degradation in BLM-induced pulmonary fibrosis, further demonstrating that SZL-P1-41 inhibited the Skp2 E3 ligase activity in the model.

Additionally, we investigated the effect of Skp2-inhibition on early phase apoptosis in the BLM model. As shown in Supplementary Figure S8, TdT-mediated dUTP nick end labeling (TUNEL)-positive cells in control mice were observed on day one, and peaked at day one after BLM treatment. The increase in TUNEL-positive cells at day three by BLM treatment was significantly inhibited in the Skp2 inhibitor-treated mice.

Moreover, we tried to determine whether TUNEL-positive cells are AECII at day three after BLM-administration. As shown in the Supplementary Figure S9, we found some TUNEL-positive cells in SFTPC-positive AECII cells (shown representatively with black squares), whereas there were also TUNEL-positive cells in SFPTC-negative uncharacterized cells (shown representatively with green squares). Some of type-I alveolar epithelial cells (AECI), determined by their morphological character (shown representatively with blue squares), seemed to be TUNEL-positive, suggesting that early apoptosis observed in BLM-induced fibrosis occurred in both AECI and AECII cells. The result is consistent with the review report by King et al. [15]. Although it is important to determine in which types of cells the apoptosis was suppressed by Skp2-inhibitor-treatment, we could not determine it because of the limitation of this method. Altogether, these results suggested that Skp2-inhibition by SZL-P1-41 as well as *Skp2*-deficiency inhibited progression of BLM-induced pulmonary fibrosis.

## 3. Discussion

In this study, pathological evaluations using Ashcroft and fibrosis scores indicated that the progression of BLM-induced pulmonary fibrosis was significantly attenuated in *Skp2*-deficient mice. Moreover, the expression of fibrosis markers such as COL1A1 and fibronectin in the lung and the accumulation of BALF cells in BLM-infused mice were also suppressed in *Skp2*-deficient mice. Furthermore, a small molecule Skp2 inhibitor (SZL-P1-41) significantly inhibited progression of pulmonary fibrosis and the accumulation of fibrosis markers. Moreover, SZL-P1-41 inhibited p27 downregulation in BLM-induced lung fibrosis indicating that SZL-P1-41 inhibited the E3 ligase activity of SCF-Skp2 in this model. These results suggested that Skp2 is involved in the progression of pulmonary fibrosis as an E3 ligase and may be a potential molecular target for IPF treatment. The CDK inhibitor p27 is the most important Skp2 target in cell cycle regulation. We have previously reported that Skp2 is involved in the proliferation of tubular epithelial cells that is required for tubule dilatation accompanied by renal injury progression in the chronic renal fibrosis model [7,12]. The increase of renal interstitial cells including fibroblastic cells by the injury was also suppressed by *Skp2*-deficiency. Skp2 may promote cell proliferation of fibroblastic cells as well as epithelial cells by degrading p27. Based on these findings, we then determined whether p27 expression was altered in BLM-induced lung fibrosis and found that p27 levels were significantly decreased by BLM-administration in WT mice, whereas p27 did not decrease in *Skp2^−/−^* mice. This result suggests that the decreased p27 expression in BLM-induced fibrosis was restored by *Skp2*-deficiency. Moreover, we performed double immunostaining experiments to identify the p27-positive cells and showed that the decrease of p27-positive cells and increase of Ki67-positive cells in mesenchymal fibroblasts by BLM-administration was suppressed by *Skp2*-deficency. The increase of mesenchymal fibroblasts by BLM-administration was suppressed by *Skp2*-deficency. These results suggested that Skp2 may participate in the BLM-induced increase of mesenchymal fibroblasts via the Skp2/p27 axis. Accumulations of COL1A1 and fibronectin were suppressed by *Skp2*-deficiency and Skp2-inhibition. It is well known that fibroblastic cells produce fibrosis-associated proteins such as COL1A1 and fibronectin. Therefore, *Skp2*-deficiency may suppress the increase of mesenchymal fibroblasts by BLM-administration and contribute to the suppression of the accumulation of fibrosis-associated proteins in lung tissues, thereby attenuating the progression of pulmonary fibrosis. Therefore, Skp2 may be involved in the progression of BLM-induced fibrosis in the late stage via Skp2/p27 axis-mediated proliferation of cells promoting fibrosis. In the early phase of BLM-induced pulmonary fibrosis, Tanaka et al. indicated that the deficiency of CHOP (CCAAT/enhancer binding protein homologous protein), an endoplasmic reticulum (ER)-stress-induced transcription factor, suppressed BLM-induced pulmonary fibrosis via inhibition of early events such as the induction of apoptosis and inflammatory-related genes and accumulation of BALF cells [16]. Therefore, CHOP participates in the progression of early phase events in BLM-induced pulmonary fibrosis. Additionally, our results suggest that Skp2 participates in late phase fibrosis and early phase apoptosis. This result is consistent with the finding that *Skp2*-deficiency suppressed apoptosis of renal epithelial and interstitial cells in a progressive kidney fibrosis model [7,12]. Even though there are many reports that Skp2 inhibits apoptosis in cancer cells, our result is contradictory. We speculate that the suppression of early apoptosis by Skp2-inhibition in the BLM-model may be an indirect effect. Skp2-inhibition may suppress early inflammation, but further studies are required to clarify this. In the early phase of BLM-induced lung injury, apoptotic cell death was found in both AECI and AECII. However, Skp2 in AECI and AECII may not directly participate in apoptosis of these cells. Skp2 may contribute to accumulation/proliferation of inflammatory cells in injured lung. Therefore, Skp2 absence/inhibition may prevent the accumulation/proliferation of inflammatory cells and apoptosis. The reduction of inflammatory cells may reduce inflammatory factors that can induce apoptosis such as TNFα, thereby apoptosis of AECI and AECII may be escaped.

Wang et al. indicated that depletion of Skp2 led to up-regulation of p27 and p21 in autoreactive pathogenic T cells (Tpaths) [17]. Skp2-repression also increases expression of Foxp3, a key transcription factor in the control of regulatory T cells (Treg) which suppresses induction and proliferation of the effector T cells [18] and induces the conversion of Tpaths into regulatory T cells (Treg) producing IL-10. In contrast, overexpression of Skp2 in Treg reduced Treg function by decreasing Foxp3 [17]. Treg produce anti-inflammatory cytokine IL-10 to suppress IL-17 expression in Th17 cells, which promotes a recruitment of neutrophils to the inflammation sites. It is reported that neutrophils firstly migrate to the lung in the early inflammation phase in the BLM-model [19]. Additionally, Park et al. reported that forced expression of Foxp3 reduced airway inflammation in a mouse asthma model via inhibiting accumulation of total immune cells, eosinophils, neutrophils, macrophages and lymphocytes neutrophil accumulation in BALF [20]. Altogether, in early inflammation of BLM-induced fibrosis, Skp2 negatively regulates Treg via suppression of Foxp3 and p27/p21. Thus, Skp2 inhibition may increase IL-10 production in Treg via accumulation of Foxp3 and p27/p21, which leads to inhibition of inflammation. Additionally, the increased Treg may also suppress IL-17 expression in Th17 cells, possibly resulting in a decrease of neutrophils accumulation in the BLM-administered lung. Skp2 inhibitors may contribute to suppression of inflammation via inhibition of apoptosis of pulmonary cells such as AECI and AECII in the early phase of BLM-model. Further studies are required to prove the speculation.

Both *Skp2*-deficiency and treatment with the Skp2 inhibitor SZL-P1-41 increased p27 levels in the BLM-infused model suggesting that Skp2 targets p27 for degradation in pulmonary fibrosis; however, it has been reported that Skp2 targets many important proteins in addition to p27, including p21, p57, p130, p300, Tob1, FOXO1, Akt, and RASSF for ubiquitin-mediated proteasomal degradation [8,9,10,11]. We cannot deny the contribution of other Skp2 targets proteins in BLM-induced pulmonary fibrosis. To determine whether p27 degradation by Skp2 is required for BLM-induced pulmonary fibrosis progression, further studies are required. Moreover, the expression of other important proteins such as α-SMA, basic-FGF and TGF-β receptor II should be determined to obtain a complete overview of BLM-induced fibrosis.

Moreover, our results suggested that the Skp2 inhibitor may be applicable for IPF treatment, because this model is a putative model of human IPF. SZL-P1-41 interferes with the formation of the SCF-Skp2 E3 ligase complex by inhibiting binding between Skp2 and Skp1. Because other SCF-type E3 ligases such as SCF-Fbw7 and SCF-β-TrCP are not inhibited [14], it is expected to specifically inhibit SCF-Skp2 E3 ligases. Additionally, several small molecule Skp2 inhibitors have been reported [21]. These include not only inhibitors of the Skp1/Skp2 interaction such as SZL-P1-41, but also inhibitors of the Skp2/p27 interaction, the Skp2/Cks1 interaction, the Skp2/p300 interaction and inhibitors of Skp2 expression. If p27 is the most important Skp2 target in pulmonary fibrosis, inhibitors of the Skp2/p27 interaction and Skp2/Cks1 interaction, which are expected to be selective inhibitors of p27-degradation, may be suitable for targeted therapy. Alternatively, if combined inhibition of the degradation of several Skp2 targets is required, inhibitors of the Skp1/Skp2 interaction or inhibitors of Skp2 expression may be effective against pulmonary fibrosis. Either way, our results suggest that Skp2 is a novel molecular target for human pulmonary fibrosis including IPF.

## 4. Materials and Methods

### 4.1. Experimental Animals

Ten-week-old C57BL/6 male mice were purchased from SLC (Shizuoka, Japan). *Skp2^+/−^* mice were mated to prepare WT (*Skp2^+/+^*), *Skp2^+/−^* and *Skp2^−/−^* mice used for these experiments [22]. All mice were given access to food and water *ad libitum* and were kept in a 12-h light/dark cycle. The mice were treated according to protocols approved by the Hamamatsu University School of Medicine Animal Care Committees at the Center Animal Care facility (approval number; 2013016, approval date: 6 July.2013; approval number: 2016019, approval date: 30 March 2016, revised date: 29 March 2017).

### 4.2. Murine Model of Pulmonary Fibrosis and SZL-P1-41 Treatment

To induce pulmonary fibrosis, mice were anesthetized with the combination of three anesthetics (0.75 mg/kg medetomidine, 4.0 mg/kg midazolam, and 5.0 mg/kg butorphanol) and treated with a single dose of 2 mg/kg BLM (Nippon Kayaku, Tokyo, Japan) [6] via intratracheal infusion on day one. Control animals were given an equal volume of saline only.

SZL-P1-41 (3-(2-benzothiazolyl)-6-ethyl-7-hydroxy-8-(1-piperidinylmethyl)-4*H*-1-benzopyran-4-one) (Aobious, Gloucester, MA, USA) [14] was dissolved in corn oil (5 mg/mL), and 10-week-old C57BL/6 WT male mice were treated with 80 mg/kg of the inhibitor or corn oil through daily intraperitoneal injection. Mice were sacrificed by cervical dislocation on day 15 after BLM or saline treatment.

### 4.3. Bronchoalveolar Lavage (BAL)

After mice were sacrificed, tracheas were cannulated using a 20-gage catheter. BAL was performed three times with 1.0 mL ice-cold saline. The BAL fluid was centrifuged, and the precipitate was collected. After total cells were counted, cells were smeared on glass slides in Cytospin preparations, fixed, and stained with Diff-Quik products (SYSMEX, Hyogo, Japan). The number of macrophages, neutrophils and lymphocytes per 400 cells was counted based on morphology.

### 4.4. Histochemical Staining

Dissected lungs were fixed in 4% paraformaldehyde (Wako Pure Chemical Industries, Osaka, Japan), embedded in paraffin, and 4-mm-thick sections were stained with HE and MT staining [23]. To examine the degree of fibrosis, microscopic images of HE-stained tissues were graded according to the Ashcroft method [13]. Moreover, bright-field images of MT staining were captured at 50× magnification, and the area of fibrosis (stained green on a red background) was quantified using ImageJ (https://imagej.nih.gov/ij/) software (fibrosis score).

### 4.5. Immunohistochemistry

To evaluate the area of fibrotic tissue, sections were then deparaffinized and rehydrated in decreasing concentrations of ethanol. Antigen retrieval was performed in a steamer using citrate buffer (pH 6.0) for 30 min. All sections were treated with fresh 3% hydrogen peroxide for 10 min at room temperature, and then washed with PBS. The sections were incubated with 10% normal blocking serum for 30 min, and then with anti-COL1A1 (ab21286, Abcam, Cambridge, UK), anti-Fibronectin (ab23750, Abcam, Cambridge, UK) or anti-p27 (610241, BD Biosciences, San Jose, CA, USA) [7]. After washing in PBS, the sections were incubated with appropriate HRP-conjugated goat anti-rabbit secondary antibody (Nichirei Biosciences, Osaka, Japan), visualized by DAB (Nichirei Biosciences, Osaka, Japan), and counter-stained with hematoxylin. The specificities of these antibodies were checked by immunoblot analysis. Anti-fibronectin and anti-p27 antibodies each detected a single band (data not shown). Anti-COL1A1 antibody detected the main band of COL1A1 and a few weak extra bands. The main COL1A1 band was increased by BLM treatment. Moreover, there was a significant correlation between COL1A1 score and fibrosis score (*r* = 0.9151, *p* < 0.0001) (Figure 2). Images of COL1A1 and Fibronectin staining were captured at 50× magnification, and the stained area was quantified using ImageJ software. Images of p27 staining were captured at 400× magnification, and cellular p27 content was semi-quantitated according the grading scale: 0, not stained: 1, slightly stained: 2, mildly stained: 3, moderately stained: 4, strongly stained. Per section, 300 cells were graded, and the grading point and the number of cells with the same grade were multiplied; the resultant value of all grades were then summed.

Double immunostaining experiments to identify type II alveolar epithelial cells (AECII) and mesenchymal fibroblasts used antibodies against surfactant protein C (SFTPC) and vimentin, respectively [24]. Paraffin-embedded lung tissues were subjected to immunostaining with anti-p27 antibody or anti-Ki67 antibody using DAB and with anti-SFTPC antibody for type II alveolar epithelial cells (AECII) or anti-vimentin antibody for mesenchymal fibroblasts using alkaline phosphatase-mediated staining. Until blocking, the same method as used for single staining was performed. After blocking, the sections were incubated with anti-SFTPC antibody (ab90716, Abcam, Cambridge, UK) or anti-vimentin antibody (sc-7557, Santa Cruz Biotechnology, Dallas, TX, USA) and then washed in PBS. The sections were incubated with secondary antibody biotin conjugated donkey anti-rabbit IgG (711-065-152, Jackson ImmunoResearch, West Grove, PA, USA) (diluted ×200 in PBS) for 2 h, and then with Streptavidin Alkaline Phosphatase (Nichirei Biosciences, Osaka, Japan), and visualized with fast red (Nichirei Biosciences, Osaka, Japan). Then, secondary staining with anti-p27 antibody or anti-Ki67antibody (RM-9106-S0, Thermo Fisher Scientific, Waltham, MA, USA) using DAB was performed as described above.

### 4.6. Statistical Analysis

Data are presented as means ± SD. Correlation with two groups were analyzed with Pearson’s product–moment correlation coefficient. Comparisons between two groups were analyzed with Student’s *t*-test. For multiple-group comparisons, one-way ANOVA followed by Tukey’s test was performed. *p <* 0.05 was considered statistically significant.

## Supplementary Materials

Supplementary materials can be found at http://www.mdpi.com/1422-0067/19/2/474/s1.

## Abbreviations

AECI	type I alveolar epithelial cells
AECII	type II alveolar epithelial cells
ANOVA	analysis of variance
BALF	bronchoalveolar lavage fluid
BLM	bleomycin
CDK	cyclin dependent kinase
COL1A1	type 1 alpha 1 collagen
DAB	3,3′-Diaminobenzidine
ECM	extracellular matrix
ER	endoplasmic reticulum
FGF	fibroblast growth factor
HE	haematoxylin-eosin
HRP	horseradish peroxidase
MT	Masson trichrome
PBS	phosphate buffered saline
PDGF	platelet-derived growth factor receptor
SCF	Skp1/Cullin/F-box
SFTPC	surfactant protein C
Skp2	S-phase kinase-associated protein 2
TUNEL	TdT-mediated dUTP nick end labeling
TGF-β	transforming growth factor
VEGF	vascular endothelial growth factor
WT	wild type
