# Supplementary material for: Inhibiting Skp2 E3 Ligase Suppresses Bleomycin-Induced Pulmonary Fibrosis
Source: Int J Mol Sci. 2018 Feb 6;19(2):474. doi: 10.3390/ijms19020474 (PMC5855696; doi:10.3390/ijms19020474)
Supplement: Supplementary file 1 [file ijms-19-00474-s001.pdf]

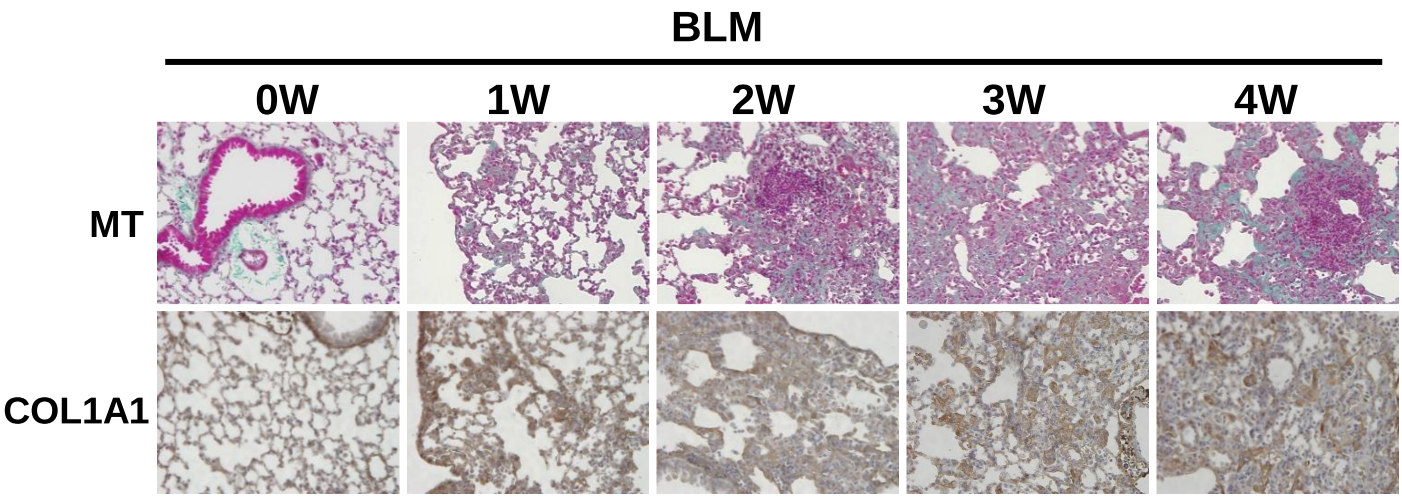


**Supplementary Figure S1. Pathological features of the BLM-induced pulmonary fibrosis model**

WT mice were intratracheally infused BLM (2 mg/kg). Mice were sacrificed, and lung tissues were harvested at the indicated weeks post-BLM-injection. Paraffin-embedded lung tissues were subjected to Masson's trichrome (MT) staining and anti-COL1A1 immunostaining.

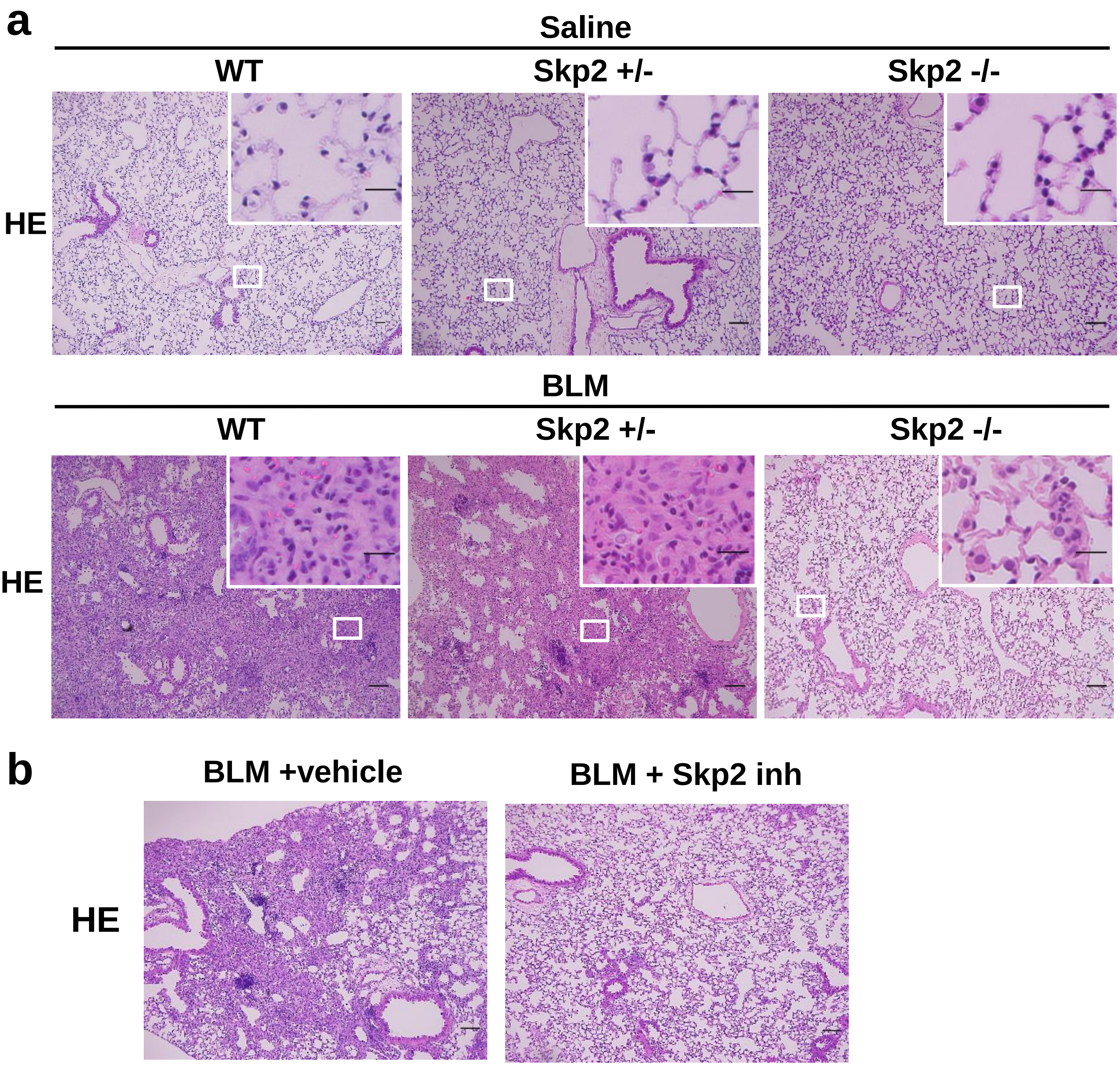


**Supplementary Figure S2. Pathological analysis of BLM-induced pulmonary fibrosis by HE staining**

**(a)** Representative images of HE-stained lungs from *Skp2*$^{+/+}$ (WT), *Skp2*$^{+/-}$ and *Skp2*$^{-/-}$ mice injected with saline (upper panels) or BLM (lower panels) from Figure 1. WT, *Skp2*$^{+/-}$ and *Skp2*$^{-/-}$ mice were intratracheally infused BLM (2 mg/kg) or saline. Two weeks after BLM treatment, the mice were sacrificed, and lung tissues were harvested. Paraffin-embedded lung tissues were subjected to HE staining. **(b)** Representative images of HE-stained lungs from BLM-infused mice treated with SZL-P1-41 or vehicle as described in Figure 5. WT mice were intratracheally infused BLM (2 mg/kg). One day after BLM treatment, SZL-P1-41 (80 mg/kg) or corn oil (vehicle) was daily injected for 13 days. Two weeks after BLM treatment, lung tissues were obtained. Paraffin-embedded lung tissues were analyzed by HE staining. The scale bar indicates 100 μm in the low magnification images and 20 μm in the high magnification images.

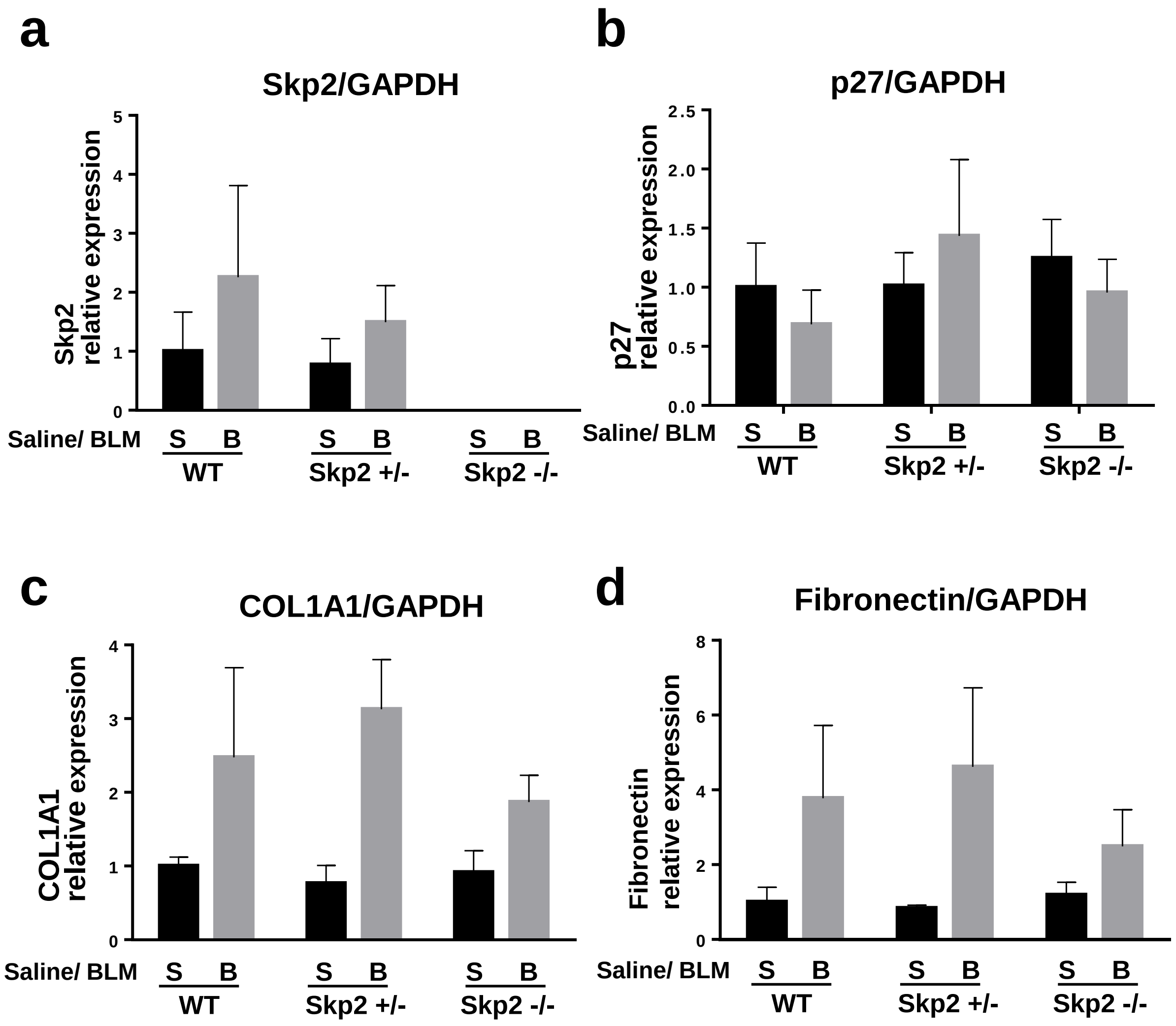


**Supplementary Figure S3**. **mRNA expression in BLM model mice.**

Total RNAs were prepared from lung tissues obtained from indicated mice 2 weeks after BLM-infusion as described in Figure 1. Indicated mRNA expressions were analyzed by RT-qPCR.

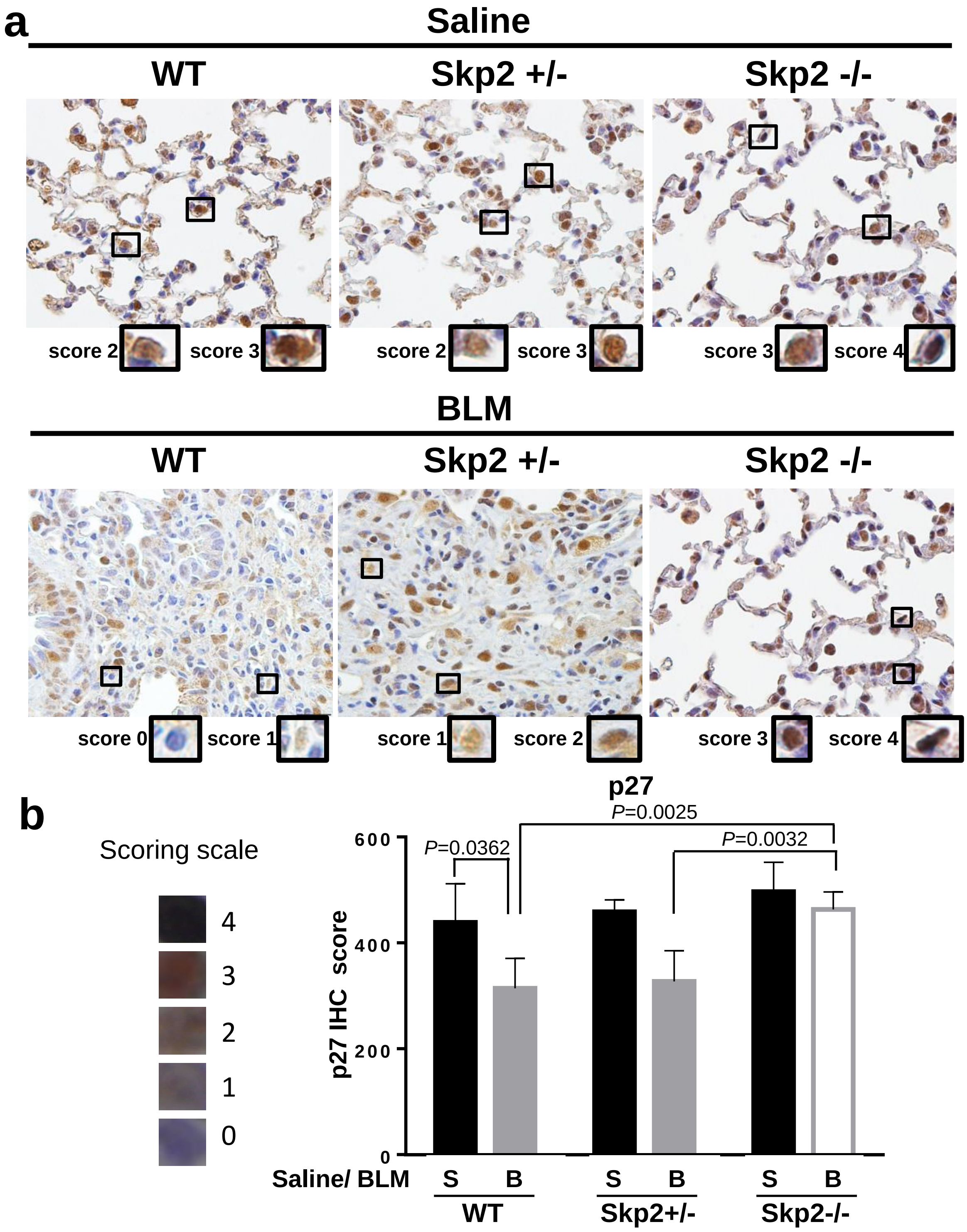


**Supplementary Figure S4. Effects of *Skp2*-deficiency on p27 levels in BLM-induced pulmonary fibrosis:**

**(a)** Representative DAB-colored images of p27 immunostaining. Paraffin-embedded lung tissues from *Skp2*$^{+/+}$ (WT), *Skp2*$^{+/-}$ and *Skp2*$^{-/-}$ mice treated with saline (upper panel) or BLM (lower panel) were subjected to immunostaining with anti-p27 antibody. The representative high-magnification images for the scoring are indicated below. The scale bar indicates 20 μm. **(b)** p27 levels were significantly decreased by BLM-treatment in WT mice but not in *Skp2*$^{-/-}$ mice. Intensities of anti-p27 immunostaining were scored as indicated intensities, and then evaluated by Tukey's test as described in the Materials and Methods.

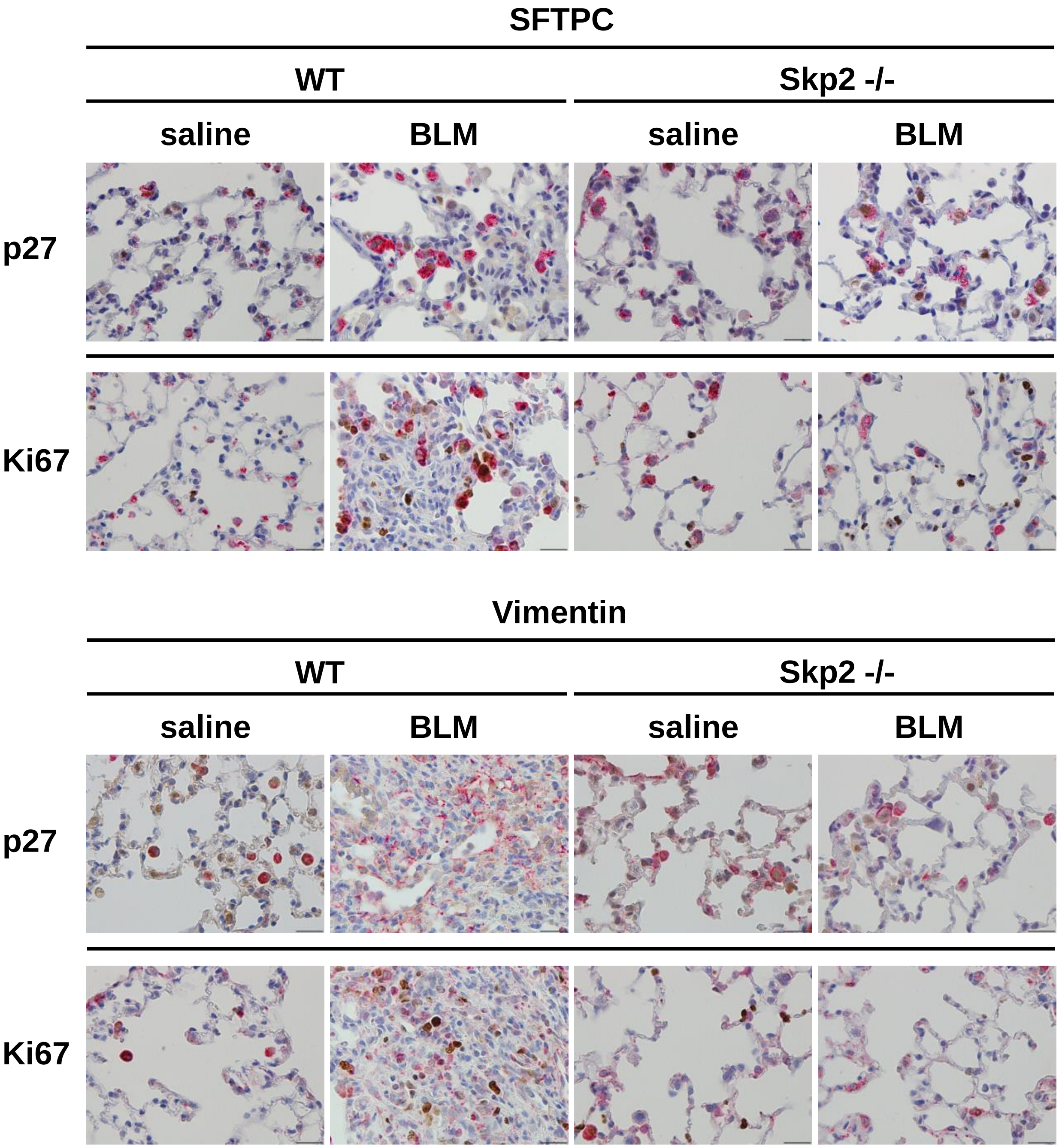


Mikamo et al. Supplementary Figure S5

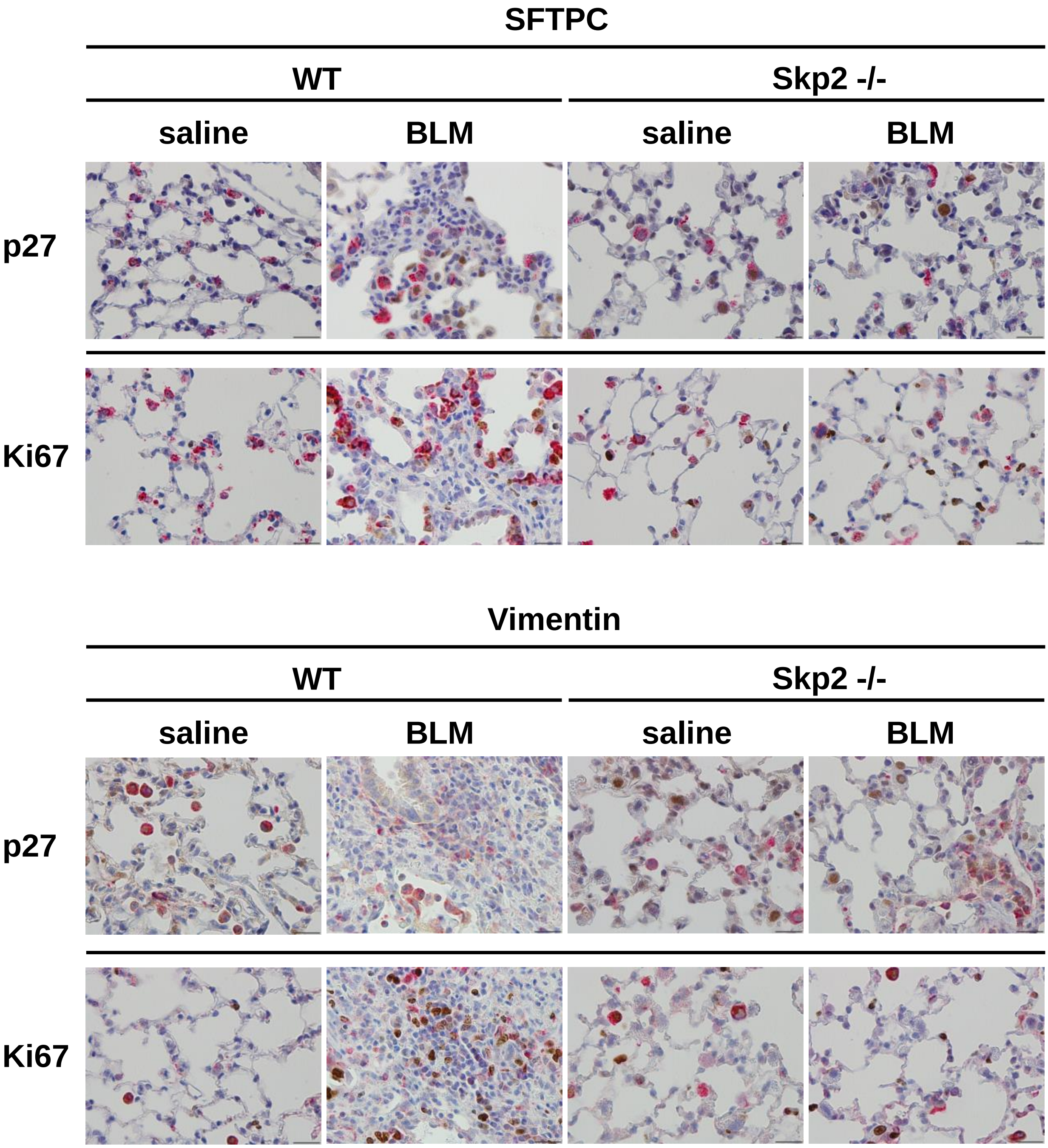


**Supplementary Figure S5**. Two additional image sets of the representative images of Figure 4

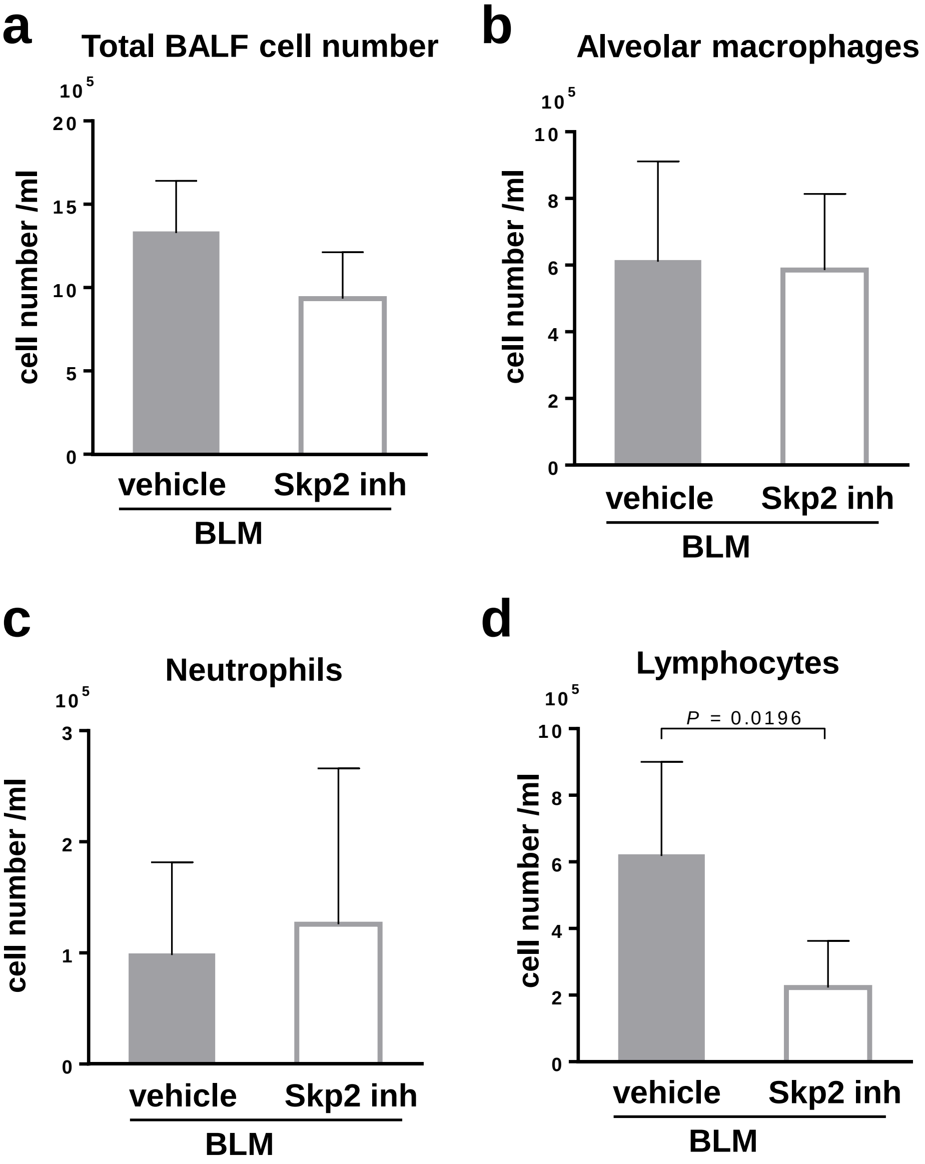


**Supplementary Figure S6. Effects of SZL-P1-41 treatment on bronchoalveolar lavage fluid cells in BLM model mice.**

BALF were obtained from mice 2 weeks after BLM-infusion as described in Figure 5. **(a)** The total number of BALF cells were counted and evaluated by Student's t-test. **(b-d)** Pulmonary alveolar macrophages **(b)**, neutrophils **(c)** and lymphocytes **(d)** in SZL-P1-41- or vehicle-treated BLM model mice were counted and evaluated by Student's t-test.

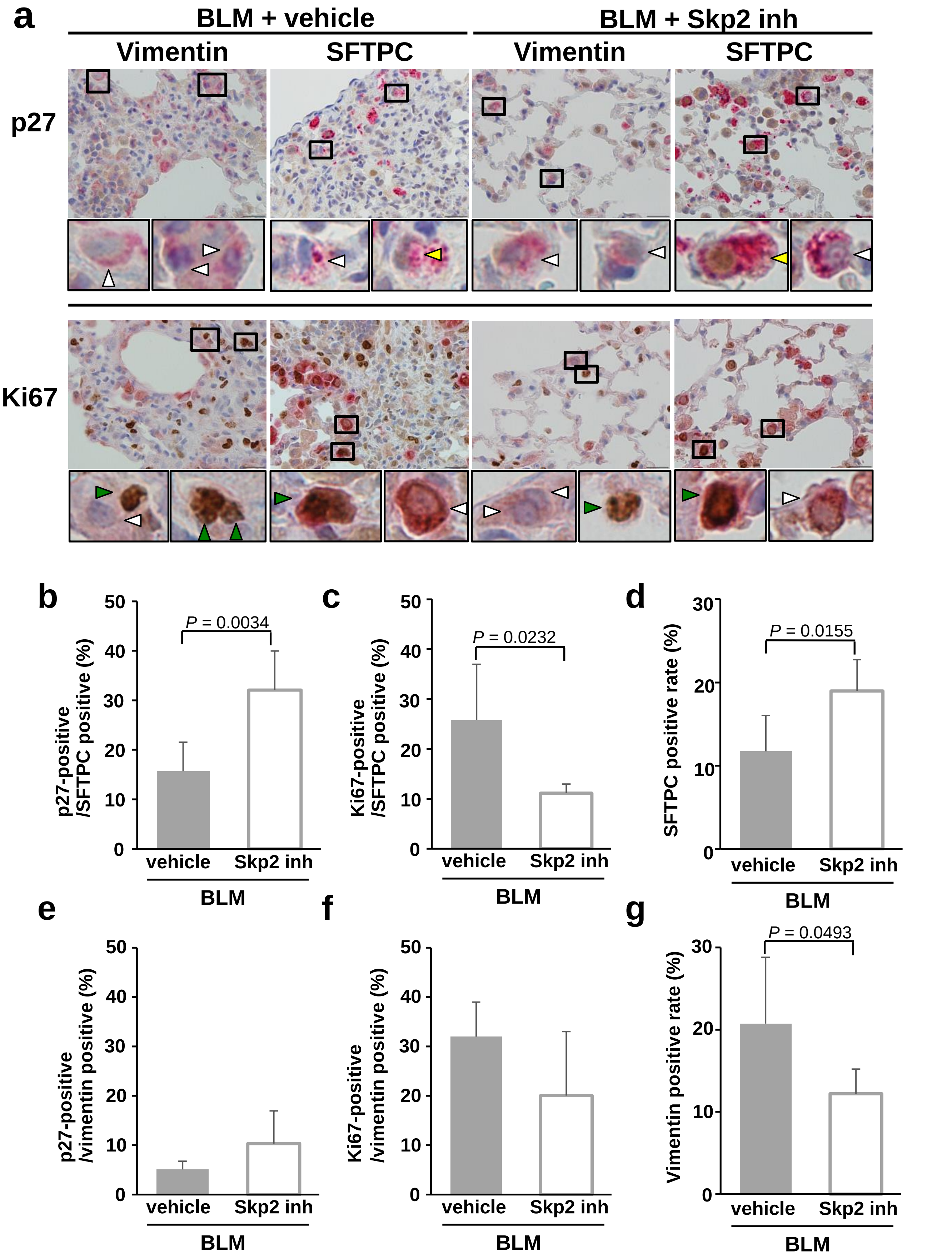


Mikamo et al. Supplementary Figure S7

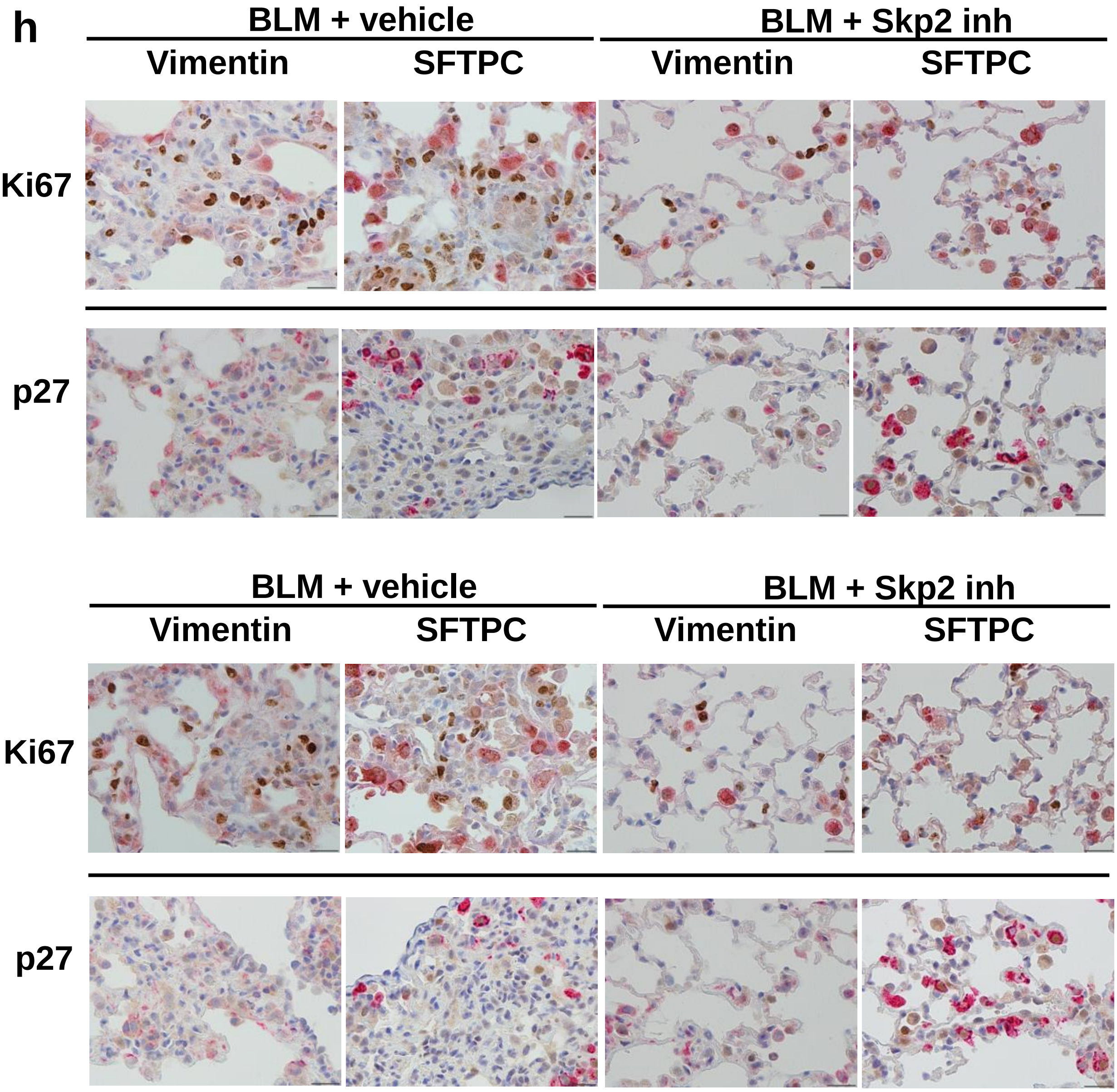


**Supplementary Figure S7. Effects of *Skp2*-inhibition on p27-expression and proliferation of type II alveolar epithelial cells and mesenchymal fibroblasts in BLM-induced pulmonary fibrosis:**

Paraffin-embedded lung tissues from BLM-administrated mice treated with vehicle or Skp2 inhibitor SZL-P1-41 as indicated in Figure 5 were subjected to immunostaining with anti-p27 antibody or anti-Ki67 antibody using DAB and with anti-SFTPC antibody for type II alveolar epithelial cells (AECII) or anti-vimentin antibody for mesenchymal fibroblasts using alkaline phosphatase-mediated staining. **(a)** Representative double immunostaining images. The representative high-magnification images are indicated below. **(b)** Ratios of p27-positive cells in SFTPC-positive cells. **(c)** Ratios of Ki67-positive cells in SFTPC-positive cells. **(d)** The abundance of SFTPC-positive AECII cells. **(e)** Ratios of p27-positive cells in vimentin-positive cells. **(f)** Ratios of Ki67-positive cells in vimentin-positive cells. **(g)** The abundance of vimentin-positive mesenchymal fibroblasts. (**h**) Two additional image sets were indicated to support the result. The scale bar indicates 20 μm.

**a**

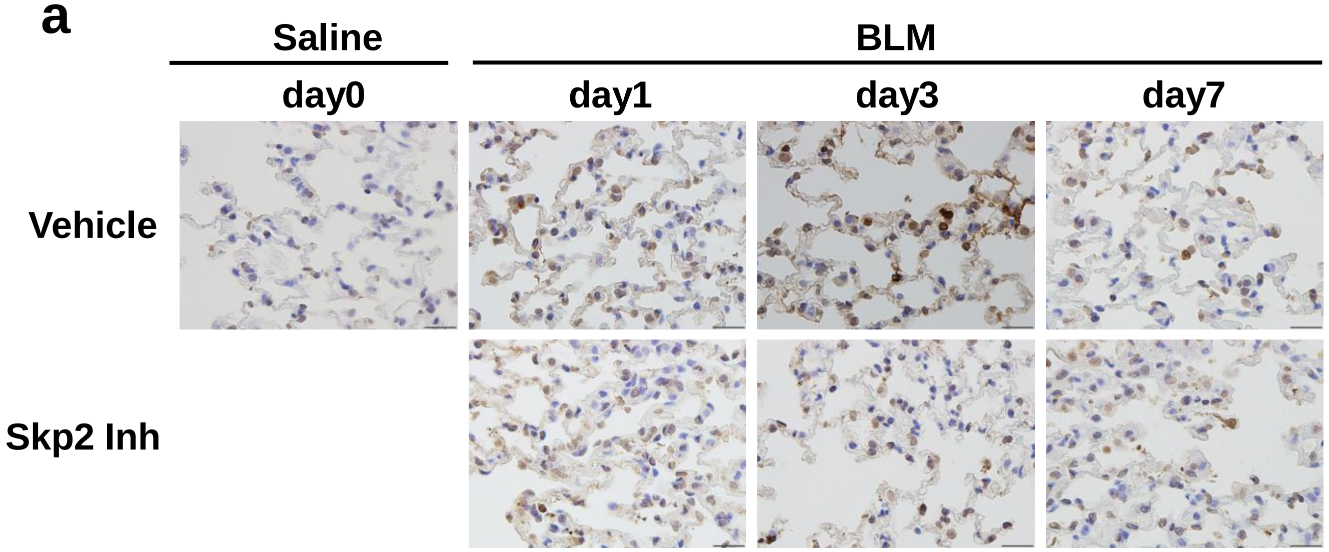


**b**

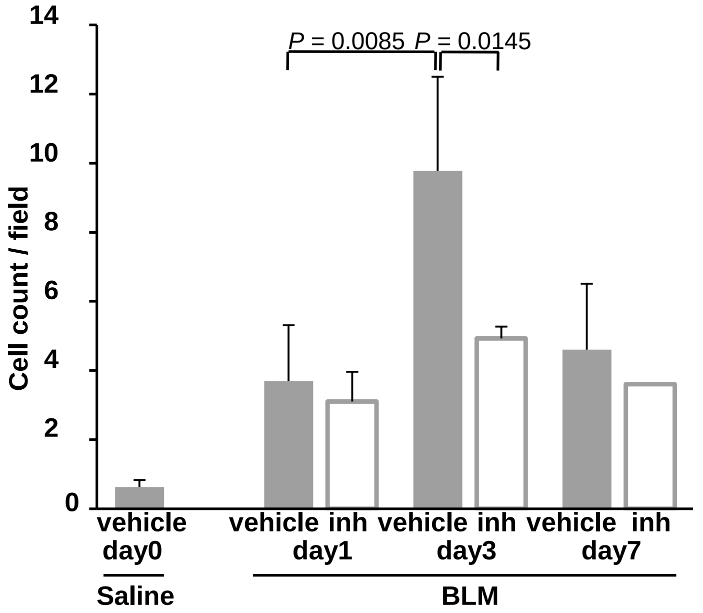


Mikamo et al. Supplementary Figure S8

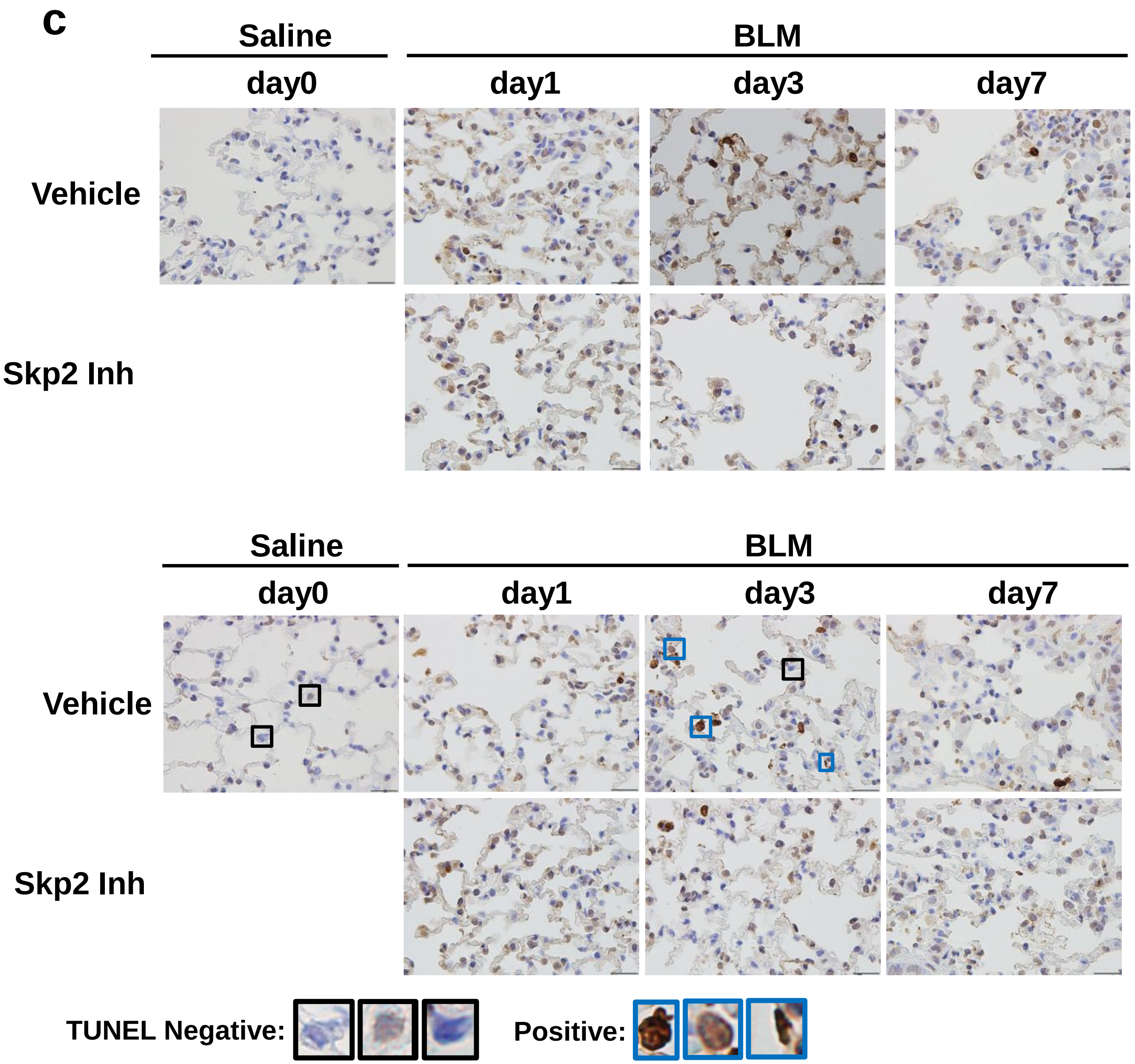


**Supplementary Figure S8. Effect of Skp2 inhibition on the early apoptosis in BLM-induced fibrosis.**

Wild type mice were intratracheally infused BLM (2 mg/kg). The Skp2 inhibitor SZL-P1-41 (80 mg/kg) (n=4) or corn oil (vehicle) (n=4) was injected intraperitoneally daily from day 1 to day 7. Indicated day after BLM treatment, lung tissues were obtained from the mice and analyzed by TUNEL assay using ApopTag (Merck Millipore). The representative images were indicated in **(a)**. TUNEL-positive cells were counted and evaluated by Tukey's test **(b)**. (**c**) Two additional image sets were indicated to support the result. The scale bar indicates 20 μm.

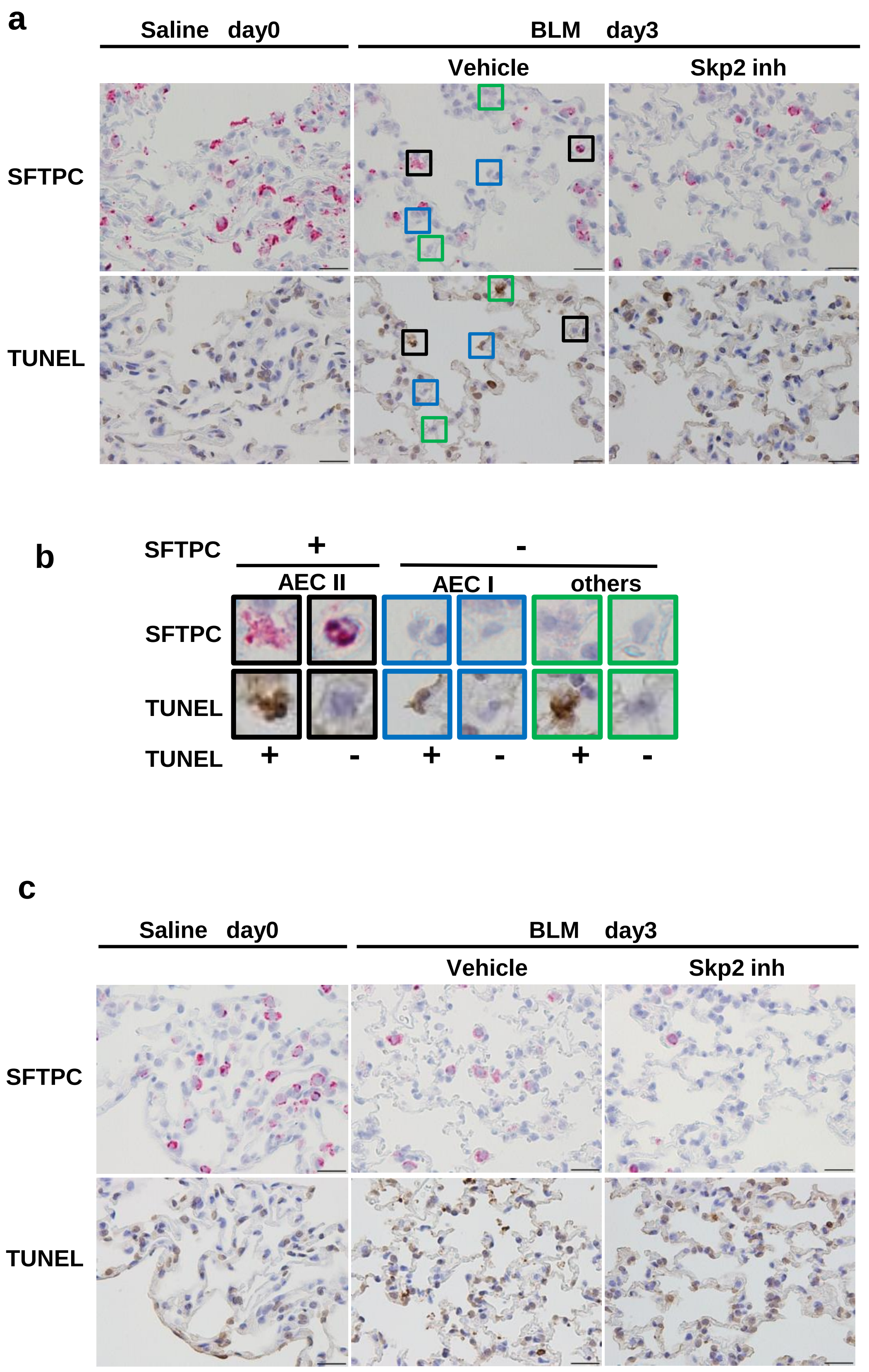


Mikamo et al. Supplementary Figure S9

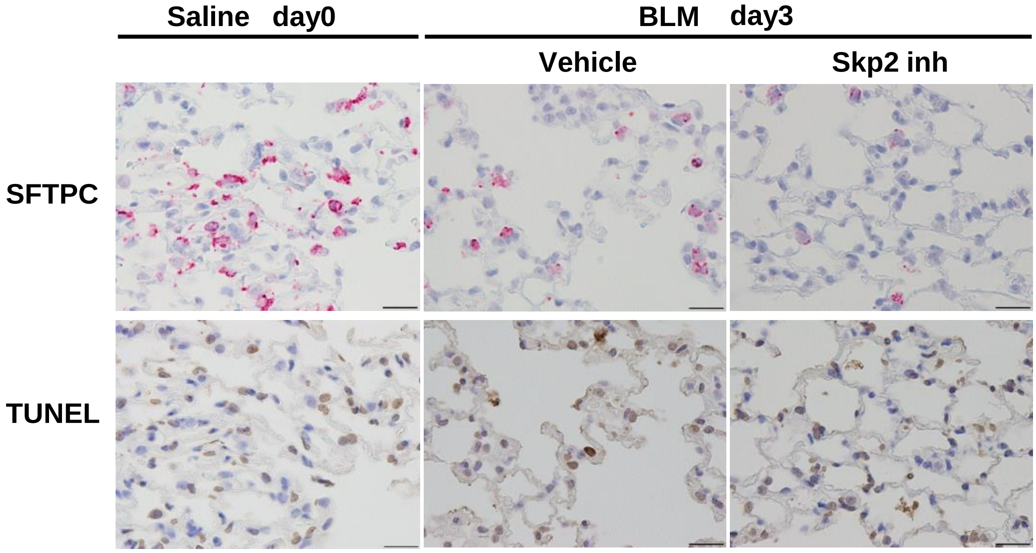


**Supplementary Figure S9. Immunostaining of consecutive lung sections with anti-SFTPC antibody to identify TUNEL-positive cell types in the early phase of BLM-induced pulmonary fibrosis.**

**(a, c)** The consecutive mirror image sections were prepared from the lung tissues using Supplementary Figure S8. These sections were subjected to TUNEL-staining and SFTPC-immunostaining, separately. (**b**) The representative high-magnification images of indicated cells. (**c**) Two additional image sets were indicated to support the result. The scale bar indicates 20 μm.
